# Assessing the validity and reliability of computational phenotyping of mood

**DOI:** 10.1371/journal.pcbi.1014597

**Published:** 2026-08-03

**Authors:** Pablo Carrillo, Marc Benhamou, Roeland Heerema, Jean Daunizeau, Mathias Pessiglione, Fabien Vinckier

**Affiliations:** 1 Motivation, Brain and Behavior Team, Paris Brain Institute/CNRS UMR 7225/INSERM U1127/Sorbonne Université, Paris, France; 2 Université Paris Cité, Paris, France; 3 Institut de Neuromodulation, Service Hospitalo-Universitaire, GHU Paris Psychiatrie & Neurosciences, Paris, France; Aarhus University: Aarhus Universitet, DENMARK

## Abstract

Mood can be understood as an affective state resulting from the integration, over time, of positive and negative outcomes. To capture intra- and inter-individual variability in mood fluctuations, computational models have been increasingly applied to self-reported mood ratings obtained during behavioural tasks. Such computational models of mood may be useful tools for understanding mood disorders. However, to be used in a clinical setting, their validity and reliability should be assessed. Recent versions of these models incorporate reciprocal interactions between mood and event perception, governed by specific parameters. Hence, it is critical to determine the extent to which estimated parameters depend on potentially arbitrary aspects of experimental design (e.g., feedback sequences) and to assess their psychometric test-retest stability. We used two widely established mood-induction tasks—a lottery task and a general-knowledge quiz—alongside a newly developed task, designed to allow precise experimental control over outcome sequences, while preserving participants’ perception that outcomes depended on their actions. Extensive numerical simulations were conducted to test the robustness of the computational models. To evaluate test-retest reliability, 163 healthy volunteers completed the tasks twice, separated by a two-week interval. Simulations demonstrated robust parameter recovery overall, though estimating the effect of mood on feedback perception proved more challenging. All tasks successfully induced mood fluctuations, accurately described by models employing leaky integration of feedback. Test-retest reliability was satisfactory for two important parameters, baseline mood and accumulated feedback weight, with significant correlations observed across most parameters. Furthermore, our newly developed task confirmed that mood-related parameter estimates remained largely unaffected by specific feedback sequences. Computational models of mood dynamics show robust validity and satisfactory test-retest reliability. Stable parameters, such as baseline mood and feedback weighting, endorse the application of these models in longitudinal studies, offering a reliable methodological basis for clinical research on mood disorders.

## Author summary

Our mood changes from moment to moment as we experience positive and negative events. Researchers increasingly use mathematical models to describe these changes and to understand why mood fluctuates differently across people. Such models could eventually help study mood disorders, but they first need to be tested carefully: do they capture real mood changes, and do they give stable results when the same person is tested again? In this study, healthy volunteers completed mood-inducing tasks twice, two weeks apart. Two tasks were already commonly used in this field, and we also developed a new task that allowed us to control the sequence of positive and negative feedback while maintaining participants’ impression that outcomes depended on their actions. We also ran numerical simulations to check whether the models could reliably recover the quantities they were designed to estimate. Overall, the models captured mood fluctuations well. Some quantities, especially baseline mood and the impact of recent feedback on mood, were reasonably stable over time. Our new task also showed that these estimates were not strongly influenced by the exact feedback sequence. These findings support the use of such models in future studies of mood and mood disorders.

## Introduction

The classic definition of mood by Jean Delay refers to “a basic affective disposition […] that gives each of our states of mind a pleasant or unpleasant tone, oscillating between the extreme poles of pleasure and pain” [1]. While emotion is thought to be closely tied to a specific event, mood is thought to reflect the cumulative effect of multiple stimuli [2–4]. How mood fluctuates in response to (or independently of) life events is a crucial question for understanding both everyday affective fluctuations and mood disorders.

Empirical observations of the effect of life events on mood fluctuations are common [5–11], in real life as well as in lab experiments [12,13], but only recently have formal theories been proposed [14–18]. An important step in this direction has been the use of computational models to analyse mood fluctuations on a short time scale, approached through a series of self-report mood ratings collected during behavioural tests [19–23]. In short, such computational model conceive mood as an affective state that integrates over time the positive and negative outcomes. Several variations have been suggested around this idea. A first group of models conceives mood as a “representation of momentum.” Mood is updated via a delta-rule driven by reward prediction error rather than direct reward values. As a result, mood converges toward the temporal derivative of reward availability in the environment. Some of these models include an effect of mood on the perception of obtained outcomes (thereby facilitating reinforcement learning [23]) and/or an effect on the perception of prospects [24–26] (thereby facilitating the decision to engage in reward-effort trade-offs). A variation on this idea is that mood is not just a representation of the external world, but rather captures an agent’s ability to achieve positive outcomes in that world [27]. In a recent study, we proposed a simple mechanism to implement this idea at the algorithmic level, dubbed the MAGNETO model [28]. In this model, mood is updated through a delta-rule using the net value of actions (reward minus cost) as the driving outcome signal. Consequently, mood converges toward a global net expected trade-off (how much rewards are expected to outweigh the time and effort invested in upcoming potential actions). Beyond integrating costs, mood becomes a representation of average reward availability rather than its temporal momentum. The critical idea underlying these models is that such a process could be adaptive in an autocorrelated environment (e.g., in early spring, the appearance of low-hanging fruits on a given tree predicts the appearance of fruits on other trees within the next few weeks, and vice versa when “winter is coming” [14,28]).

Experimentally, several tasks have been proposed to induce short-term (i.e., < 2 hours) mood changes. The most frequently used are gambling/lottery tasks [19], in which participants receive positive and negative feedback as a result of a random draw. The outcome could be completely independent of the agent’s will (as in a wheel of fortune), or it could depend on the agent’s choice to participate in the lottery (e.g., a choice between 1 euro for sure or a 50% chance of winning 2 euros). More recently, we and others have used a quiz task designed to induce episodes of high correct response rate (leading to higher mood) vs. low correct response rate (leading to lower mood) [24–26]. The idea behind this task was to maximize the sense of agency, which might arguably be minimal in a lottery task where participants could rely on simple decision heuristics. The underlying hypothesis is that mood might be more responsive to outcomes when they depend on the agent’s behaviour, than when they depend solely on the environment [29–31]. However, this increased sense of agency comes at the cost of reduced experimental control: the course of the task, including the sequence of outcomes, inherently depends on participants’ choices and responses rather than being fully determined by the experimenter. This reduced control may affect the validity and reliability of inferred computational phenotypes.

Studies using these tasks have demonstrated that positive and negative feedback can induce measurable changes in mood. These mood fluctuations were then modelled in terms of a leaky accumulation (weighted sum across time, where the weight exponentially decays with delay) of outcomes and/or expected outcomes. Some models added a reciprocal effect of mood on outcome perception, such that good mood induces a “rosy outlook”. Given that individuals may integrate identical feedback sequences differently (leading to distinct mood trajectories), these models include free parameters that characterize the temporal dynamics of mood responses to past outcomes. Critically, reciprocal interactions make the model nonlinear: the current mood state influences how subsequent feedback is integrated, so small differences in early event sequences can produce markedly different long-term trajectories. This raises an important methodological concern: to what extent do the free parameters derived from participants’ mood ratings genuinely reflect stable individual traits, rather than being driven by incidental differences in the feedback sequence they experienced?

A promising application of computational models of mood is the phenotyping of patients with mood disorders, either to better understand the cognitive mechanisms underlying the emergence of mood swings, to identify their neurobiological correlates, or to develop clinical tools for predicting patient outcomes [32–35]. However, longitudinal studies in patients with neuropsychiatric disorders require measures with established test-retest reliability. When reassessing the same patient after a therapeutic intervention, it is essential to determine whether observed changes reflect meaningful cognitive changes rather than measurement variability. When using computational modelling, reliability must be considered at two complementary levels. First, model reliability can be assessed using simulation and recovery analyses [36], which involve generating synthetic data with predefined parameters, fitting the model to the data as if they were real data, and evaluating the deviation between generative and fitted parameters. Second, behavioural reliability can be assessed through model-free measures and computational parameters.

**Fig 1:**
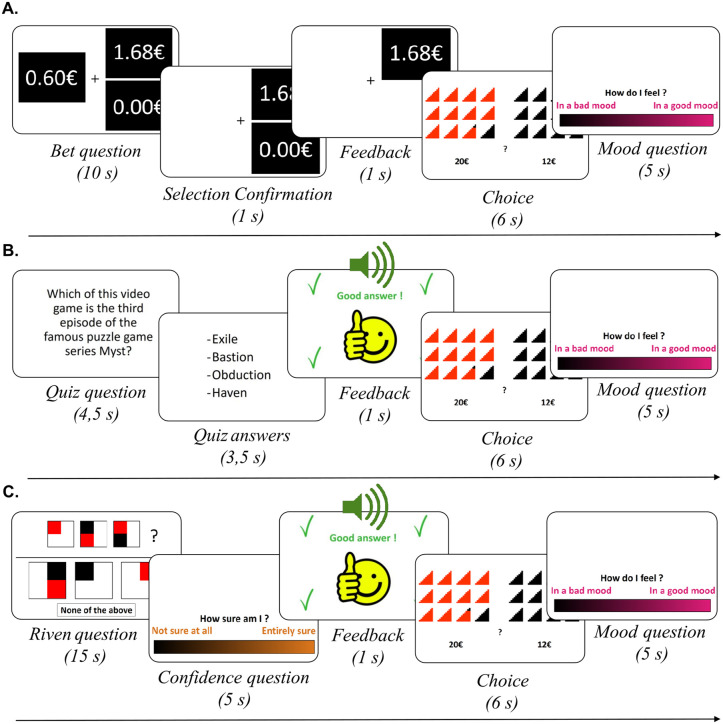
Task presentation. **(A)** Lottery task: Participants chose between a certain gain/loss and a gamble with equal probabilities of €0 or a larger gain/loss. **(B)** Quiz task: Participants answered general knowledge questions, with biased difficulty and feedback meant to generate episodes with high and low correct response rates. **(C)** Riven task: Participants solved puzzles by selecting the most logical item to complete a series. The design allowed for a fixed sequence of feedback across participants. In all tasks, feedback was followed by an economic choice involving effort, delay, or risk. Each trial ended with a subjective mood rating. The smiley clip-art is an open-source image and can be found online (https://openclipart.org/detail/321098/thumbs-up-smiley).

The objective of this study is threefold. First, we aim at evaluating how the free parameters controlling short-term mood fluctuations depend on the sequence of feedback used. For this purpose, we developed a new paradigm that allowed us to externally control the sequence of feedbacks while participants falsely believed that the outcomes depended on their responses. This new task was compared to previous tasks used in the literature (namely, the lottery task and the quiz task, see methods and Fig 1). Second, we aim at evaluating the robustness of the mood models used in the literature, both in terms of model identification and parameter recovery. For this purpose, we used a simulation and recovery approach. Third, we aim at assessing test-retest consistency by repeating the same task twice, two weeks apart.

## Results

### Simulations

#### Procedure.

We first tested the robustness of a representative set of candidate models previously used in the literature to fit mood rating timeseries. To assess model identifiability and parameter recovery, we used a simulation-recovery approach. From the model’s perspective, the three tasks differed only in the nature of the outcome variable: continuous in the lottery task and binary in both the quiz and the Riven task. For each type of outcome (continuous or binary), 200 synthetic participants were simulated by sampling parameter values from uniform distributions that were chosen to yield plausible behaviours. For each model variant and two sequences of 75 feedbacks, a series of mood ratings was simulated. We then fitted the mood ratings generated under each model to assess model identifiability (i.e., the extent to which the model that generated the data was identified by the model selection procedure) and parameter recovery (i.e., the extent to which fitted parameters correlated with simulated parameters when the same model was used to generate and fit the data). In the model identifiability analysis, we performed random-effects Bayesian model comparison and computed each model’s exceedance probability (denoted xp); we report the mean, across both sequences, of this exceedance probability for each model variant. In the parameter recovery analysis, we compared simulated and fitted parameters and report the mean Spearman correlation, across both sequences, for each alternative model variant. Since a common practice in the literature is not to ask for a mood rating on every trial, but rather to assess mood every few trials, we also varied the frequency of mood ratings available by downsampling mood ratings, i.e., removing one-fourth, one-third, or one-half of the generated mood ratings. Finally, we evaluated whether or not linearly interpolating mood ratings was a valid strategy for compensating missing data points. Note that only simulations with binary outcomes are reported here (with only minor differences found with continuous outcomes, S1 Fig).

### Model space

All candidate models were variants of previously published models in which mood is generated by integrating tasks events over time, as follows:


$$\begin{array}{c} m_{\left(t\right)}={\omega_{0}+\;\omega }_{f}\sum_{j=1}^{t}\gamma^{t-j}*f_{\left(j\right)}+\omega_{e}\sum_{j=1}^{t}\gamma^{t-j}*{EV}_{\left(j\right)}+\;\omega_{t}*t \end{array}$$

where *m*_*(t)*_ denotes the predicted mood at time or trial *t*, *f* denotes perceived feedback, and *EV* denotes the expected value of the outcome. The free parameters *γ* and all *ω* are estimated from the data at the subject level: *ω*_*0*_ represents a baseline mood term, *ω*_*f*_ the weight assigned to accumulated feedback, *ω*_*e*_ the weight assigned to expected value, and *ω*_*t*_ a linear effect of time. The parameter *γ*, with 0 < *γ* < 1, is a forgetting factor that controls the relative influence of recent events versus more distant events.

Since mood ratings were expressed on a bounded scale from -1–1, *m*_*(t)*_ was transformed using a sigmoid function. The inverse temperature parameter *c* was fixed a priori to avoid collinearity with the weight parameters *ω,* which determine the scale of *m*_*(t)*_.


$$\begin{array}{c} m_{r(t)}=(\frac{\text{1}}{\text{1}+e^{\left(-c*m_{\left(t\right)}\right)}})*\text{2}-\text{1} \end{array}$$

We generated a set of candidate models by systematically varying the following specifications:

- We allowed for an asymmetric influence of positive and negative events on mood, using a rescaling parameter *a* (with *a* > 0) for positive outcomes:

$$\begin{array}{c} \begin{array}{cc} f_{\left(t\right)}=\;a*{out}_{\left(t\right)} & {out}_{\left(t\right)}>\text{0}\\ f_{\left(t\right)}=\;{out}_{\left(t\right)} & {out}_{\left(t\right)}<\text{0} \end{array} \end{array}$$

where *out*_*(t)*_ denotes the outcome at trial *t*.

- As previously suggested in the literature, we also allowed mood to influence the subjective perception of feedback, as follows:

$$\begin{array}{c} f_{\left(t\right)}={out}_{\left(t\right)}+\delta \cdot m_{\left(t-\text{1}\right)} \end{array}$$

where *δ* is a scaling free parameter that controls the effect of mood on perceived feedback.

- We also tested a simplified version of the model in which only the outcome (or its subjective perception *f*) was considered, meaning that *ω*_*e*_ was fixed to 0.

All combinations of these specifications yielded a total of 8 model variants (Table 1). Two “control” models were also added to the model space: one in which mood was a linear function of trial number (*m(t) = ω*_*0*_ *+ ω*_*t*_*·t*) and one in which only the last feedback was considered (*m(t) = ω*_*0*_ *+ ω*_*f*_*·f*_*(t)*_ *+ ω*_*t*_*·t*), equivalent to a forgetting factor *γ* fixed to 0.

**Table 1:** Model space. Two control models and 8 models of interest were studied in terms of model identifiability and parameter recovery.

Model Number	Model Name	Asymmetry between negative and positive outcomes (a)	Reciprocal effect of mood on feedback (δ)	Impact of expected value on mood ($\omega_{e})$	Original publication
M1	Control1	Control model where mood is a linear function of time			
M2	Control2	Control model where only the last feedback is considered			
M3	Asy-Rec-Exp-	0	0	0	
M4	Asy-Rec-Exp+	0	0	1	Rutledge et al. 2014
M5	Asy-Rec + Exp-	0	1	0	
M6	Asy-Rec + Exp+	0	1	1	Eldar & Niv 2015, Eldar et al. 2016
M7	Asy + Rec-Exp-	1	0	0	
M8	Asy + Rec-Exp+	1	0	1	
M9	Asy + Rec + Exp-	1	1	0	Vinckier et al. 2018
M10	Asy + Rec + Exp+	1	1	1	

This model space is conceived as a summary of previous research focusing on explicit mood modelling in laboratory-based studies. In a landmark study, Rutledge and colleagues introduced the leaky accumulation model of affect integrating outcomes, expected values and reward prediction errors [19]. Eldar and colleagues added a reciprocal effect of mood on outcome perception, first in a multiplicative [23], then in an additive form [14]. We introduced an asymmetric effect of negative and positive outcomes in a previous study [24], as well as a time drift as a nuisance term. Notably, a negative drift in mood during such tasks has since been shown to be a robust contextual effect [37].

### Model and parameter recovery

**Fig 2:**
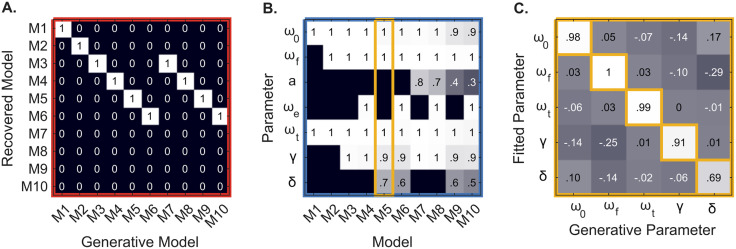
Model and parameter recovery for noiseless ratings. Mood ratings are entirely reliable and available at every trial, for a total of 75 trials. (A) Confusion matrix (exceedance probability of each model given the model that generated the data). (B) Overview of same-parameter recovery for every model. Every cell displays the correlation between a generative parameter and the corresponding recovered parameter when the same model is fitted. (C) Parameter recovery for the selected model. Cross-parameter correlation matrix when the same model is fitted. The diagonal corresponds to the fifth column of the middle panel.

As a baseline, we first examined model and parameter recovery using noiseless ratings. Model identifiability was almost perfect for all models that did not include a parameter introducing asymmetry between positive and negative feedback weight (all xp > .99, Fig 2A). By contrast, models including an asymmetry between positive and negative outcomes were essentially non-identifiable. Specifically, the simpler model without asymmetry was consistently identified as the best-fitting model, irrespective of whether the mood ratings were generated by a model including an asymmetry term. Further analyses showed this lack of identifiability reflected a complex interaction among several factors, rather than simple model overparametrisation (S3 Appendix).

Parameters *γ* and all *ω* were accurately recovered for all models (all *r* > .86, all 95%CI lower bounds > .82, Fig 2B and S2 Fig). The remaining two parameters, *δ* and *a*, showed less accurate recovery, with estimated correlations between simulated and fitted parameters between 0.32 and 0.65.

### Effect of rating reliability

In order to assess the effect of noisy mood ratings, we incorporated a reliability parameter *α* controlling the relative contribution of the model-predicted signal and random response variability. Specifically, after mapping the latent model prediction onto the bounded response scale, the observed response was generated as a mixture of this bounded signal and a random contaminating component:


$$\begin{array}{c} y_{t}=\alpha \;m_{r(t)}+(\text{1}-\alpha )\epsilon_{t} \end{array}$$

where ϵ_t_ was sampled from a uniform distribution over the same interval, ϵ_t_ ∼ U (−1,1), and *α*∈ [0,1]. This formulation ensures that simulated observations remain within the admissible response range for all values of *α*. The parameter *α* can be interpreted as a reliability or signal-preservation parameter: when *α* = 1, responses are fully determined by the model-predicted signal, whereas when *α* = 0, responses are independent of the model and uniformly distributed over the response scale. Intermediate values correspond to partial corruption of the model-predicted signal by bounded random variability.

**Fig 3:**
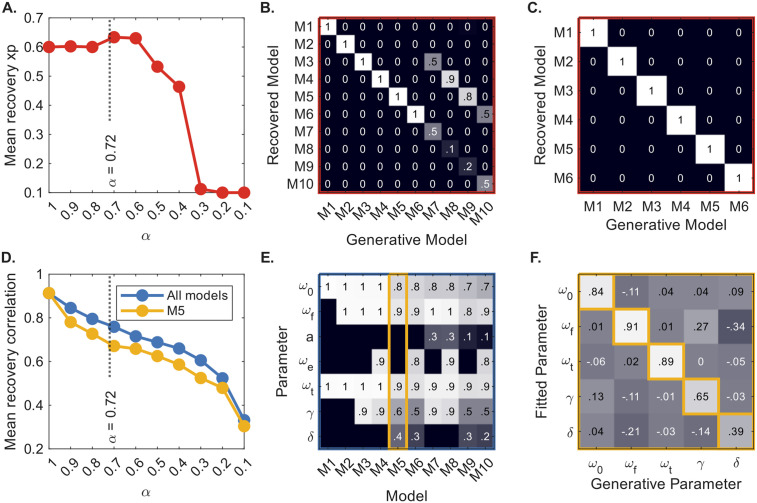
Effect of mood rating reliability on model and parameter recovery. (A) Mean model recovery exceedance probability as a function of decreasing signal weight. (B) Confusion matrix (exceedance probability of each model given the model that generated the data) of the full model space for the experimental noise estimate (α = 0.72). (C) Confusion matrix of the restricted model space for the experimental noise estimate. (D) Mean parameter recovery correlation as a function of decreasing signal weight. (E) Same-parameter recovery across models for the experimental noise estimate (α = 0.72). (F) Selected model cross-parameter correlation for the experimental noise estimate.

Systematically assessing model and parameter recovery with varying *α* values, we found that global model identifiability plateaus until *α* values around 0.5 (corresponding to a signal-to-noise ratio of 1), before decreasing sharply. By contrast, parameter recovery follows a seemingly linear decrease until *α* values around 0.3 before collapsing (Fig 3A and 3D).

To assess the global reliability of mood ratings in the experimental dataset, we estimated the empirically predictable variance using a global mixed-effects linear model that included lagged Volterra predictors and autoregressive terms for past mood ratings. This analysis yielded a R2 of 0.865, corresponding to an estimated reliability parameter *α* = 0.717, with the remaining unexplained variance taken as a plausible estimate of empirical noise.

Examining confusion matrices around this value, we found that the model recovery pattern remained close to the noiseless reference, with perfect identification of models that didn’t include an asymmetry parameter (all xp > .99, Fig 3A-3C). Consistent with the global pattern, parameter recovery was more impacted by noise than model identifiability at the empirical estimate. Recovery remained high for *ω* parameters (*r* > 0.67, 95%CI lower bound > .59), but fell to intermediate levels for the decay factor *γ* (0.51 < *r* < 0.94), which became sensitive to the presence of the parameter that governs the reciprocal effort of mood on feedback perception, *δ* (Fig 3D-3F). The two remaining parameters *a* and *δ* showed low recoverability (*r* < .40, 95%CI higher bound < .50).

### Effect of rating number, frequency and interpolation

**Fig 4:**
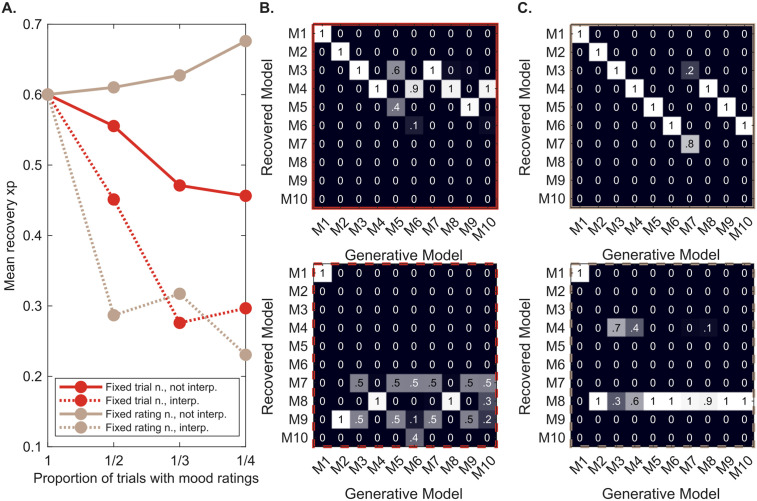
Effect of the number and frequency of mood ratings on model identifiability. (A) Mean recovery exceedance probability as a function of the proportion of trials in which mood ratings are collected, in two situations: fixed total trial number (red) and fixed total mood rating number (grey pink). Dotted lines denote linear interpolation for missing data. (B) Confusion matrix (exceedance probability of each model given the model that generated the data) when mood ratings are collected every 4 trials out of a total of 75 trials, without (top) and with (bottom) linear interpolation for missing data. (C) Confusion matrix when mood ratings are collected every 4 trials for a total of 75 mood ratings (300 trials), without (top) and with (bottom) linear interpolation for missing data.

When mood ratings were downsampled, simulating a design in which mood was not probed on every trial, model identifiability decreased significantly. Specifically, the identifiability of the model variants that included an effect of mood on feedback perception (*δ*) was impaired, as their simpler variant (without this reciprocal effect) was selected by model comparison. However, keeping the number of mood ratings constant while decreasing their frequency tended to improve model recovery, suggesting that the observed decrease in model identifiability was attributable to the smaller number of samples rather than to lower rating frequency per se (Fig 4). We also noted that, for a fixed number of mood ratings, model recovery was strongly determined by the temporal coverage of the rating schedule. Uniformly spaced ratings outperformed unconstrained random sampling, likely because random schedules produced clusters and long gaps that reduced observability of the latent mood trajectory, and are therefore presented as the main results (but see S3 and S4 Figs for results with random sampling).

Importantly, linear interpolation, often used in the literature to compensate for missing mood ratings, systematically decreased model identifiability, such that only Control Model 1 was correctly identified (Fig 4B, bottom row). Surprisingly, linear interpolation biased model recovery toward more complex models (M7 and M9), even when the data were generated by very simple ones. These results were preserved across interpolation methods, suggesting that impaired recovery was a general consequence of the interpolation procedure rather than a consequence of the specific interpolation method used.

**Fig 5:**
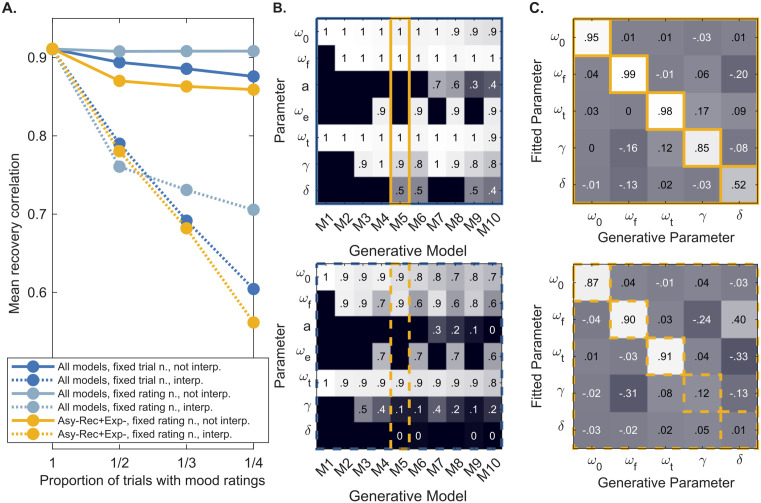
Effect of the number and frequency of mood ratings on parameter recovery. (A) Mean parameter recovery correlation as a function of the proportion of trials in which a mood rating is collected, in two situations: fixed total trial number (blue) and fixed total mood rating number (grey blue). Dashed lines denote linear interpolation for missing data. (B) overview of same-parameter recovery for every model. Every cell displays the correlation between a generative parameter and the corresponding recovered parameter when the same model is fitted, without (top) and with (bottom) linear interpolation for missing data. (C) Cross-parameter correlation matrices for the selected model, fitted on native only (top) or linearly interpolated (bottom) ratings. Diagonals correspond to the highlighted columns in the middle panel.

Parameter recovery was moderately affected by mood-rating downsampling, with the average recovery correlation decreasing from  .91 to  .86 when only a quarter of mood ratings were retained (Fig 5A-5B). One parameter whose recovery was particularly affected was the reciprocal-effect parameter, *δ*. For example, in model 5 (Asy-Rec + Exp-), its recovery correlation decreased from  .65 (95%CI, [.57,  .73]) to  .52 (95%CI, [.41,  .61]).

As for model identification, keeping the number of mood ratings constant while lowering their frequency tended to improve parameter recovery, suggesting that the observed decrease was attributable to the smaller number of samples rather than to lower rating frequency per se (Fig 5A).

Likewise, using linear interpolation to compensate for missing mood ratings systematically impaired parameter recovery, a result that once again did not depend on the interpolation method. This reduction affected all parameters, with the most pronounced effects observed for *δ* and *γ* (Fig 5B-5C, bottom row).

### Behavioural data

Below, we describe the results of three tasks designed to induce mood fluctuations. Those three tasks, referred to as the “lottery task”, the “quiz task” and the “Riven task”, were conducted on 54 participants for the lottery task (mean age 37.44 years, standard deviation 16.58 years, proportion of female participants 57%), 109 participants for the quiz task (mean age 34.70 years, standard deviation 15.74 years, proportion of female participants 75%) and 138 participants for the Riven task (mean age 36.54 years, standard deviation 17.18 years, proportion of female participants 67%), twice with a two weeks interval. The lottery task and the quiz task were previously used in the literature, while the Riven task was created for this study.

All three tasks involve negative and positive feedbacks designed to induce mood fluctuations. The time course of a trial is presented in Fig 1 for each task. The lottery task involves a choice between a certain gain/loss and a gamble with equal probabilities of €0 or a larger gain/loss. The quiz task involves answering general knowledge questions with biased question difficulty and feedback, in order to generate episodes of high and low correct response rates. The Riven task involved solving puzzles by determining which of three proposed items was the most logical to follow a series of three other items. Crucially, all three proposed items could be the most logical depending on the participant’s interpretation of the series, allowing the experimenter to use the same fixed sequence of feedback for each participant. This feature was included to assess whether computational phenotyping results depend on the feedback sequence. In order to be able to compute a prediction error, a subjective confidence rating was included in each trial before the feedback was given. In all three tasks, the feedback was followed by an economic choice involving effort, delay, or risk. The main purpose of these economic choices was to assess the effect of mood on decision making (see [25] for the results, where the analysed dataset includes only the quiz task). Therefore, they are not analysed further in this paper. Finally, a subjective mood rating was required on each trial in all three tasks.

### Model-free analyses

**Fig 6:**
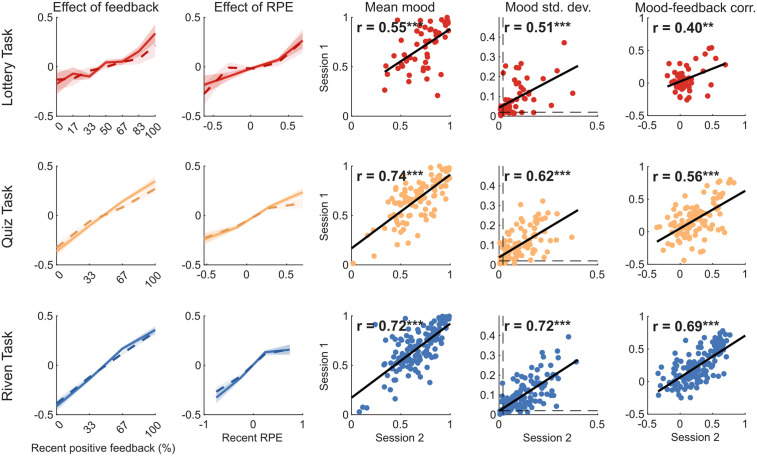
Model-free properties of mood ratings across tasks. Each row presents the results for one of the three tasks*. First column:* z-scored mood as a function of the proportion of positive feedback in the last three trials. Shaded areas represent the standard error of the mean. *Second column*: same analysis with RPE substituted for feedback. *Third and fourth columns*: correlations of the mean and standard error of mood between sessions. Mood ratings are scaled between 0 and 1. Dotted line represents our exclusion criteria for model-based analyses. *Fifth column*: correlations of mood-feedback correlation coefficients between sessions. (* *p* < .05, ** *p* < .01, *** *p* < .001).

We first verified the extent to which outcome affected mood in each of the three tasks (Fig 6). To do so, we correlated each participant’s z-scored mood with the average reward over the last 3 trials (monetary outcome in the lottery task, binary feedback in the other two tasks). The mean correlation between z-scored mood and proportion of good feedback (window of 3 previous trials) was *r* = 0.082 (95%CI [.04,  .12], *t*(53) = 3.9, *p* < .001) for the lottery task, *r* = 0.21 (95%CI [.16,  .26], *t*(104) = 9.0, *p* < .001) for the quiz task and *r* = 0.24 (95%CI [.20,  .28], *t*(137) = 12.0, *p* < .001) for the Riven task. The correlation coefficients were higher in the quiz task and Riven tasks compared to the lottery task (both *p* < .001; quiz/lottery: *t* = 4.50, df = 316; Riven/lottery: *t* = 5.84, df = 382), while there was no difference between these two tasks (*p* = .26, *t* = 1.12, df = 484). Substituting RPE to reward yielded the same result, albeit with lower correlation coefficients: there were significant correlations between RPE and mood ratings in the lottery (*r* = 0.063, 95%CI [.026, 1.0]), quiz (*r* = 0.14, 95%CI [.11,  .18]), and Riven task (*r* = 0.16, 95%CI [.13,  .20]); this correlation was lower in the lottery task than in the quiz (*t*(316) = 3.63, *p* < .001) and Riven task (*t*(382) = 3.92, *p* < .001), while there was no difference between these last two tasks (*t*(484) =  .95, p = .34). Therefore, even though the average correlation between feedback and mood was quite low, all three tasks robustly induced mood fluctuations such that higher mood ratings followed positive feedback. In the lottery task, bet acceptance rates depended on the gamble type (one-sample t-tests, all p < .001): mean ±SD in the loss, mixed and gain trials were respectively  .37 ± .24,  .53 ± .23, and  .70 ± .24.

Because most participants completed the task twice, we were able to assess the stability of descriptive model-free measures of mood fluctuations across sessions. Specifically, we examined test-retest correlations across participants for the mean and variance of mood ratings, as well as for the correlation between feedback and mood examined above. For the lottery task, the between-session correlation was 0.55 (95%CI [.33,  .71], *p* < .001) for mean mood, 0.51 (95%CI [.28,  .68], *p* < .001) for mood standard deviation, and 0.4 (95%CI [.15,  .60], *p* < .01) for the correlation between feedback and mood. For the quiz task, the between-session correlation was 0.74 (95%CI [.64,  .81], *p* < .001) for mean mood, 0.62 (95%CI [.49,  .73], *p* < .001) for mood standard deviation, and 0.56 (95%CI [.41,  .68], *p* < .001) for correlation between feedback and mood. For the Riven task, the between-session correlation was 0.72 (95%CI [.63,  .79], *p* < .001) for mean mood, 0.72 (95%CI [.63,  .79], *p* < .001) for mood standard deviation, and 0.69 (95%CI [.59,  .76], *p* < .001) for the correlation between feedback and mood (Fig 6). In addition, there was no significant difference between the first and second sessions for any of these 3 indicators in any of the tasks (all *p* > .05). Overall, these model-free analyses suggest that participants’ behaviour was stable across the two sessions, with little evidence for an effect of retesting. We therefore proceeded to the model-based analyses, which constituted the main objective of the study. In these analyses, we excluded participants with very low variability in mood ratings, defined as a standard deviation below 2%, because their mood reports provided little variance for the models to explain.

### Model-based analyses

For each participant and each experimental session, every candidate model was fitted to the time series of mood ratings. Economic choices, that did not include any feedback, were not included in the main analysis (S4 Appendix). Since models with an asymmetric effect of positive and negative outcomes were not correctly identified in the simulation-recovery analyses, we excluded them from the model space, which ultimately included 6 models (4 models of interest and two “control” models). While the expected value of the feedback was directly computable in the lottery task, this was not the case for the other two tasks. For the quiz task, the expected value was estimated as the proportion of correct answers in a previous sample of participants, while for the Riven task, we used confidence ratings as a proxy for expected value.

First, we ensured that there were no differences in model frequencies between groups (i.e., tasks) or sessions, using dedicated Bayesian tests [38]. Indeed, we found strong evidence that the distribution of model frequencies was the same in all three task-groups (xp > .99 for each session). Similarly, the distribution of model frequencies was the same in both sessions in each of the three groups (all xp > .99). Thereafter, model comparisons will be presented using session 1 results. As there was no evidence for a difference in model frequencies between groups, we ran a Bayesian model selection on all groups pooled together. The selected model was Model 5 (Asy-Rex + Exp-), i.e., the model in which only feedback was considered (without any effect of expected value), with a reciprocal effect of mood on feedback perception (xp > .99).

We then checked the proportion of participants in which the model identified as the best one was actually better than our control model 1, in which mood is just a linear function of time (Control1). This was the case for 54.1% of participants in the lottery task, for 70.0% of participants in the quiz task and for 68.8% of participants in the Riven task. There was no difference in those proportions across tasks (*χ*^*2*^ (2, N = 236) = 3.34, *p* = 0.19).

**Fig 7:**
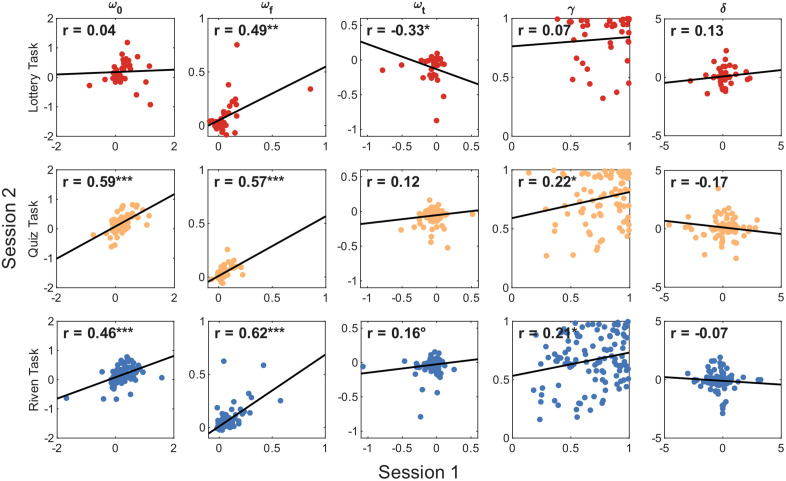
Parameter stability. Scatter plot of fitted free parameter values for sessions 1 vs. session 2 for the three tasks. Parameter values are relative to mood ratings scaled between -1 and 1 and a fixed sigmoid observation mapping. (°*p* < .1, * *p* < .05, ** *p* < .01, *** *p* < .001).

Next, we assessed the stability of model parameters across sessions. For each of the three tasks and each of the five parameters of the selected model, we computed test-retest correlations across participants, relating individual parameter estimates from the first session to those from the second session (Fig 7). In the lottery task, we found a significant correlation only for *ω*_*f*_ (*r* = 0.49, 95%CI [.20,  .70], p = .002). For the quiz task, the correlation reached significance for *ω*_*0*_ (*r* = 0.59, 95%CI [.43,  .71], *p* < .001), *ω*_*f*_ (*r* = 0.57, 95%CI [.41,  .70], *p* < .001) and *γ* (*r* = 0.22, 95%CI [.01,  .41], *p* = .04). Finally, it reached significance for the Riven task for *ω*_*0*_ (*r* = 0.46, 95%CI [.30,  .60], *p* < .001), *ω*_*f*_ (*r* = 0.62, 95%CI [.49,  .72], *p* < .001), *γ* (*r* = 0.21, 95%CI [.03,  .38], *p* = .02) and marginally so for *ω*_*t*_ (*r* = 0.16, 95%CI [-.03,  .33], *p* = .1). We then tested for difference between these correlation coefficients by transforming the correlation coefficients to z-scores using Fisher’s *r* to *z* transformation. We found differences between the lottery and the quiz task for *ω*_*0*_ (*p* = .002) and *ω*_*t*_ (*p* = .02), as well as between the lottery and the Riven task for *ω*_*0*_ (*p* = .02) and *ω*_*t*_ (*p* = .01), while between the quiz task and the Riven task we found no significant difference (all *p* > .1). Importantly, paired t-tests showed no difference between sessions for any parameter in any of the tasks (all *p* > .1).

Fitted parameter mean and standard deviations are reported in Table 2. The forgetting factor *γ* differed between tasks (*F*(2,99) = 5.98, *p* = .004); specifically, post-hoc comparisons indicated *γ* was lower in the Riven task than in both the quiz task (*p* = .004) and lottery task (*p* = .02). The reciprocal-influence parameter *δ*, which quantifies the effect of mood on feedback perception, did not differ between task (*F*(2,83) = 2.31, *p* = .11) and its global group mean did not differ from zero (*t*(235) = -1.11, *p* = .27). The weight on feedback accumulation, *ω*_*f*_, also showed a main effect of task (*F*(2,64) = 7.32, *p* = .001) with lower values in the quiz task than in the Riven task (post-hoc comparison, *p* = .003).

**Table 2:** Summary statistics of fitted parameter distributions in each task (first session). Reported figures are mean (standard deviation), for each parameter and each task. All values are relative to mood ratings scaled between -1 and 1 and a fixed sigmoid observation mapping.

Parameter	$\omega_{0}$	$\omega_{f}$	$\omega_{t}$	γ	δ
Interpretation	Mood baseline	Sensitivity to outcomes	Time drift	Forgetting factor	Reciprocal effect of mood on outcome perception
Lottery	0.25 (0.39)	0.060 (0.15)	-0.046 (0.20)	0.78 (0.19)	0.10 (0.93)
Quiz	0.20 (0.29)	0.032 (0.047)	-0.036 (0.12)	0.78 (0.20)	0.016 (1.34)
Riven	0.20 (0.34)	0.063 (0.089)	-0.056 (0.14)	0.69 (0.23)	-0.21 (0.85)

**Fig 8:**
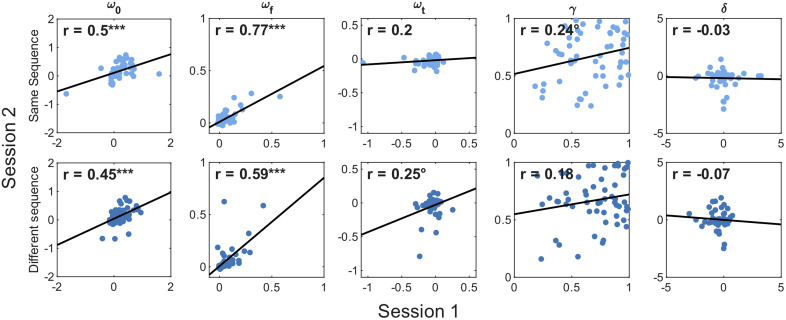
Parameter stability in the Riven Task. Scatter plot of fitted free parameter values in the Riven task. Each line denotes a separate condition: same or different feedback sequence between sessions. Parameter values are relative to mood ratings scaled between -1 and 1 and a fixed sigmoid observation mapping. (° *p* < 0.1, * *p* < .05, ** *p* < .01, *** *p* < .001).

An important aspect of the Riven task is that it allows the experimenter to control the feedback sequence. In this study, we used two feedback sequences to define two conditions: half of the participants completed both sessions with the same sequence, whereas the other half completed each session with a different sequence. This design allowed us to assess the extent to which parameter stability depended on sequence identity, which could occur if the estimated parameters were partly driven by the specific feedback sequence used (Fig 8). As in previous analyses, the correlation between sessions was significant for both subgroups for both *ω*_*0*_ (*r* = 0.50, 95%CI [.28,  .68], *p* < .001 and *r* = 0.45, 95%CI [.22,  .64], *p* < .001 respectively in the same and different sequence condition) and *ω*_*f*_ (*r* = 0.77, 95%CI [.64,  .86], *p* < .001 and *r* = 0.59, 95%CI [.39,  .74], *p* < .001 respectively in the same vs different sequence condition). Correlation did not reach significance for the other parameters, with *γ* showing a marginally significant correlation when the same sequence was used (*r* = 0.24, 95%CI [-.02,  .48], *p* = .07) and *ω*_*t*_ when different sequences were used (*r* = 0.25, 95%CI [-.01,  .48], *p* = .06). Fisher’s *r* to *z* transformation showed no difference between these correlations. Finally, we tested whether these groups had any influence on parameter values, and found an isolated group-session interaction for *δ* (*F* (1,110) = 6.15, *p* = .015), indicating opposing session-wise changes in *δ* in the two groups, with a decrease in the same-sequence group and an increase in the different-sequence group.

Finally, for each parameter and task, we quantified parameter stability as the log ratio of within-subject to between-subject variance (Table 3). This index provides a measure of the reliability of the parameter estimates relative to inter-individual variability: lower values indicate that within-subject variability is small compared with between-subject differences. Only two parameters showed greater between-subject than within-subject variability, corresponding to a negative log-ratio: baseline mood, *ω*_*0*_, and the sensitivity to feedback accumulation, *ω*_*f*_. This pattern was consistent across tasks, with the exception of baseline mood in the lottery task.

**Table 3:** Within-subject vs. across-subject variability summaries. Log-ratios of variance of means to mean of variances is reported for each computational parameter and task (with additional per-condition values for the Riven task), together with bootstrap standard error. A value of 0 indicates equal within-subject and between-subject variability. A value of 1 indicates within-subject variability was twice as high as between-subject variability, whereas a value of −1 indicates that it was half as high.

Parameter	*ω*_*0*_	*ω*_*f*_	*ω*_*t*_	*γ*	*δ*
Lottery	0.86 (0.80)	-0.56 (0.34)	2.01 (0.86)	0.79 (0.47)	0.59 (0.32)
Quiz	-0.95 (0.30)	-0.89 (0.39)	0.66 (0.43)	0.35 (0.36)	1.45 (0.46)
Riven (overall)	-0.39 (0.36)	-1.05 (0.64)	0.58 (0.28)	0.37 (0.27)	1.21 (0.16)
Riven (same sequence)	-0.46 (0.53)	-1.53 (0.45)	0.78 (0.29)	0.26 (0.35)	1.07 (0.16)
Riven (different sequence)	-0.30 (0.42)	-0.82 (0.94)	0.31 (0.49)	0.46 (0.41)	1.36 (0.28)

For the Riven task, we computed both an overall ratio and condition-specific ratios, providing a summary of how changing the feedback sequence affected relative parameter stability. The results indicate that changing the sequence reduced parameter reliability, with lower log-ratio values in the same-sequence condition for all parameters except the time drift parameter *ω*_*t*_.

## Discussion

In this study, we evaluated the internal validity and psychometric reliability of a behavioural-computational paradigm in which mood fluctuations are induced using behavioural tasks and analysed with a dedicated class of computational models. Using extensive numerical simulations, we first assessed the identifiability of different models previously used in the literature [14,19,24] and showed that some of them were indeed not identifiable. We also showed that while some free parameters were easy to recover, such as baseline mood and the weight of feedback accumulation on mood, others were much harder to recover, such as the forgetting factor or the reciprocal effect of mood on feedback perception. Importantly, the scarcity of mood ratings may worsen model identifiability and parameter recoverability, especially when interpolation is used to compensate for missing data points. Using a test-retest approach, we then assessed the stability of model-free behaviour and model-based free parameters. We identified which parameters appear to be stable after two weeks (at least in healthy participants) and which are not. Finally, using a novel mood induction task, we demonstrated that the stability of free parameters seems to be little impacted by the feedback sequence.

Model and parameter recovery is a critical sanity check in computational modelling [36]. Surprisingly, we found that mood models containing an asymmetry between positive and negative outcomes are not recoverable, while the asymmetry term itself is difficult to recover. This result is disconcerting because many reinforcement learning models include such an asymmetry in their structure [39–41]. Dedicated analyses showed that the identifiability of this parameter depends primarily on the absolute magnitude of the asymmetry, but also interacts with other model specifications, such as the inclusion of expected value accumulation and reciprocal effect of mood (S3 Appendix). One way to circumvent this identifiability issue could be to use mixed lotteries in which gains and losses are weighted in the same trials, allowing an indifference point to be fitted directly to choices and an asymmetry parameter to be inferred from it, that could in turn inform a mood model. Perhaps less surprising is the fact that the parameter that captures the reciprocal effect of mood on feedback perception, *δ*, is among the least recoverable ones. In fact, the effect of *δ* on mood dynamics can be partially reproduced by increasing the forgetting factor (thus taking more events into account). Intuitively, this can be related to the fact that *δ* introduces an autoregressive term into the mood updating rule.

Our behavioural results showed that all three tasks were able to induce mood fluctuations. They all have strengths and weaknesses. The lottery task, while the simplest, is subject to simple heuristics and does not induce a sense of control, but it has the advantage of allowing the computation of a normative expected value of feedback. The quiz task increases the sense of control, but loses the computation of the expected value and does not allow for perfect control of the feedback sequence. The Riven task, which was designed to avoid this shortcoming, allows for perfect control over the feedback sequence and thus for more fine-grained mood manipulations than the previously used tasks. Although this feature was intended to optimize the test-retest stability of computational phenotyping stability if the results depended on the feedback sequence, our results suggest that it is not crucial, as within- and across-sequence parameter correlations were comparable. Another important aspect of the Riven task is that participants can decide to invest more or less (cognitive) effort on each trial to solve the puzzles, which was not possible in previous tasks. This type of feature can be relevant to probe the influence of additional cognitive variables such as effort on mood fluctuations. The objective of this study was not to determine which task is objectively “best” for investigating mood, as this choice largely depends on the specific research questions authors aim to address. However, if anything, tasks involving a sense of control (e.g., the quiz and the Riven tasks) appear somewhat more effective, particularly regarding the proportion of participants whose mood ratings are slightly better explained by a complete model rather than a control one, and the relative stability of free parameters across sessions.

At a more general level, all the tasks used in this study consider mood fluctuations in a stable environment, in the absence of explicit learning: the outcomes experienced by participants may have motivational value, but they do not carry relevant information to learn about the task. While this approach is the most frequently used in the literature, one line of work focuses on interactions between learning-relevant variables and mood [21,23]. In particular, the reciprocal effect of mood on feedback perception has primarily been found when big singular outcomes were added to a task, arguably leveraging a learning mechanism [23]; it was conceptualised to be relevant in a context where mood interacts primarily with learning [14]. Using tasks where learning about outcomes is a key dimension might therefore lead to different findings. One can speculate that when made relevant by active learning, associated variables such as expected values and RPEs might carry more relative weight in driving mood fluctuations, potentially leading to the selection of a different model. Likewise, different models that consider an effect of mood on subjective outcome in a learning context might yield different results regarding the recoverability and stability of the associated parameters. More generally, these different empirical approaches relate to different conceptualisations of the function of mood as driving primarily reinforcement learning or motivation [28].

Interestingly, the best-fitting model of mood was consistent across all three tasks. Aside from the asymmetry between positive and negative outcomes, which was excluded from the model space, it mirrors the model previously supported by behavioural data from the quiz task [24]. Specifically, it is a model that considers only feedback (excluding the prediction error about feedback) but includes a reciprocal effect of mood on feedback. This finding contrasts with most of the existing literature, which typically integrates the effect of expected values or prediction errors into mood models. For instance, the existing literature suggests that reward prediction error, rather than feedback alone, influences mood-related processes, such as gambling tendencies in lottery tasks and the content of language on social media [10,11]. Regarding the quiz task, this discrepancy may be due to potentially poor estimates of the expected value of feedback. Since participants’ prior knowledge cannot be directly assessed, the expected value of feedback was approximated by using the average proportion of correct responses obtained from a separate group. However, at the individual level, this approach is likely inaccurate, as we assume participants have a more binary assessment of whether or not they know the answer to a specific question. A similar limitation applies to the Riven task, where the expected value of feedback was derived from subjective confidence ratings. However, the same model was also selected for the canonical lottery task, further deviating from findings commonly reported in the literature. We currently lack a robust hypothesis explaining our consistent inability to replicate the role of expectation in mood fluctuations. Aside from a potential issue of statistical power, the most plausible explanation relates to a significant alteration in our experimental design, notably the introduction of an orthogonal task (economic choice) between feedback and mood assessment. This modification was intentionally implemented to weaken the direct association participants might perceive between feedback and subsequent mood evaluation. Another significant difference from the published design is the inclusion of mood ratings on every trial, which may have narrowed the participants’ focus to the immediately preceding trial and interacted with the way outcomes and prediction errors were accumulated.

From a computational psychiatry perspective, subject-level model parameter estimates, often referred to as “computational phenotype”, are key outcomes. A central hope in the field is that these estimates will enable researchers to shed a new light on inter-individual differences, diagnosis and clinical prediction [34,42]. In this regard, it is necessary to quantitatively assess the sources of variation in parameter estimates. One potential source is the particular task used for model fitting and parameter estimation. Our results across three different tasks were mixed. We found that in the Riven task, the forgetting factor *γ* was lower than in the lottery and quiz tasks, indicating that fewer previous trials contributed to mood at a given trial. Notably, the weight of mood on subjective feedback *δ* did not differ across tasks, but was widely distributed across participants, with a group mean not significantly different from zero. Positive values of *δ* are indicative of a mood-congruent bias, resulting in a “rosy outlook” when mood is high and a “gloomy outlook” when it is down, and might promote mood swings by amplifying small fluctuations. Negative values of *δ*, by contrast, can be interpreted as a contrast effect, with higher mood leading to an attenuation of the positive valuation of better outcomes, and might reflect a tendency towards regulation around an equilibrium.

Comparing computational parameter estimates with previous literature is challenging, given that values are always relative to other model specifications, which is why few studies actually report them. This is especially true for feedback sensitivity, which is often estimated in models that also include accumulation terms for EV and RPE, making comparison with our selected model irrelevant. However, we note that baseline mood values reported in [43] are similar to our findings, with a mean around 60% of scale range. Forgetting factor values are reported in many studies that mainly use variants of the lottery task; these reported values are in the same range as our estimates [19,21,44–48]. One study also reports a time drift parameter between -2% and -4% of scale range, similar to our finding [48]. More generally, this result is consistent with previous findings that a negative time drift is a consistent and robust driver of mood during cognitive tasks [37].

Finally, we were able to assess the stability of model parameter estimates over two weeks, which is a crucial prerequisite for the use of computational phenotyping in longitudinal studies with patients suffering from neuropsychiatric disorders. The two parameters for which we found strong test-retest stability are the mood baseline *ω*_*0*_ and the weight of feedback on mood *ω*_*f*_. It is worth noting that the effect of mood on decision-making—although not reported here—has previously been demonstrated to exhibit stable properties across sessions in the same dataset [25]. Conversely, we found little to no correlation across sessions for the forgetting factor *γ* and the weight of mood on feedback perception *δ*. This finding may appear somewhat disappointing, given these two parameters are closely related to quantities that could be directly estimated using a model-free approach. Indeed, we found no evidence for a greater stability of computational fitted parameters over model-free statistics such as the mean or standard deviation of mood ratings. Importantly, we must acknowledge that since we recruited only healthy volunteers for this study, we assumed that their mood state would not vary over a two weeks interval. However, a clear limitation of our study is that we didn’t collect clinical questionnaires to verify that this was indeed the case. Therefore, the weak test-retest consistency observed for most parameters may reflect not only noisy behaviour or intrinsically limited parameter recoverability, but also genuine changes in these parameter values over timescales of days or weeks. The extent to which mood fluctuations observed at the timescale of minutes are actually relevant for understanding clinical mood swings, which often last for weeks or months [49], remains an open question that calls for longitudinal studies in healthy volunteers and patients with mood disorders.

## Methods

### Ethics statement

The study was approved by the Ethics Committee for Biomedical Research of the Pitié-Salpêtrière Hospital (AP-HP hospital group, Paris), where the experiments were conducted. All the experiments followed the guidelines and regulations of the Paris Brain Institute, and were in accordance with the Declaration of Helsinki.

### Participants

Participants were recruited using e-mail through an online database of candidates for behavioural experiments in the greater Paris area. Inclusion criteria were: being 18 years of age or older, being a French native speaker, and not having any history of psychiatric or neurologic disorders. The study took place in the PRISME behavioural facility of the Paris Brain Institute. Participants gave written informed consent before starting the experiment. They were paid a fixed endowment of €25 for each experimental session in which they participated.

A total of 163 healthy volunteers participated in this study. All participants were screened for a history of neurological or psychiatric disorders and excluded if present. 25 participants completed a first experiment comparing the quiz task with another task that is not discussed here because it did not meet its objectives (mean age 30.44 years, standard deviation 4.98 years, female proportion 80%). 84 participants took part in a second experiment comparing the quiz task with the Riven task. Out of these 84 participants, 80 completed both tasks and 4 completed only the Riven task due to technical problems or time constraints (mean age 35.96 years, standard deviation 17.53 years, female proportion 74%). Finally, 54 participants took part in an experiment comparing the canonical lottery task to the Riven task (mean age 37.44 years, standard deviation 16.58 years, female proportion 57%).

For the model-based analyses, we excluded participants whose mood ratings were not variable enough on a task-by-task basis. Specifically, we excluded participants whose mood ratings had a standard deviation of less than 2%. We also excluded participants who answered the Riven question in less than 3 seconds on average, considering that they didn’t try to find the good answer. The first exclusion criterion lead to the exclusion of 17.1% of participants (18/105) from the quiz task analysis, 15.2% of participants from the Riven task analysis (21/138), and 31.5% of participants (17/54) from the lottery task analysis. The second exclusion criterion lead to the exclusion of 5.1% of participants (7/138) from the Riven task. In total, 18.8% of Riven task participants (26/138) were excluded. Chi-squared tests with Yates correction were not significant for a difference in the proportion of exclusions between tasks (X² = 3.48, p = 0.06 between quiz and gambling, X² = 2.88, p = 0.09 between Riven and gambling, X² = 0.03, p = 0.86 between quiz and Riven).

### Tasks

Participants were given three tasks designed to induce mood fluctuations. The lottery task, adapted from [19], the quiz task, adapted from [24], and a newly developed task, the Riven task (Fig 1). The stimuli were presented using the Psychophysics Toolbox Version 3 (Psychtoolbox-3) in MATLAB R2021a (Mathworks Inc) [50–52]. In all three tasks, trials ended with a mood rating screen, where participants had to place a cursor along a continuous scale to answer the question “How do I feel?” with anchors “in a bad mood” and “in a good mood”.

### Lottery task

After 10 training trials to familiarize participants with the task and each question, participants completed 150 trials. There were three types of trials: 50 gain trials (a certain gain or an equal probability lottery between 0€ and a larger gain), 50 loss trials (a certain loss or an equal probability lottery between 0€ and a larger loss), and 50 mixed trials (a certain amount of 0€ or an equal probability lottery between a gain and a loss amount). If participants failed to respond within the 10 s time limit, they received the worst outcome. The certain option proposed a loss between -0.6 and 0€ in loss trials, a gain between 0.6 and 0€ in gain trials, or 0€ in the mixed trials. The lottery option proposed a gain between 0 and 3€ or a loss between 0 and -3€. More precisely, in the mixed trials there were 5 amounts of gain in centimes of euros (30, 50, 80, 110, 150) and the amount of loss was determined by 10 multipliers on the amount of gain (0.2, 0.3, 0.4, 0.52, 0.66, 0.82, 1, 1.2, 1.5, 2). In the gain trials, there were 5 certain amounts (20, 30, 40, 50, 60), and the lottery gains were determined using 10 multipliers on the certain amount (1.68, 1.82, 2, 2.22, 2.48, 2.8, 3.16, 3.6, 4.2, 5). In the loss trials there were 5 certain amounts (-20, -30, -40, -50, -60) and the lottery losses were determined using 10 multipliers on the certain amount (1.68, 1.82, 2, 2.22, 2.48, 2.8, 3.16, 3.6, 4.2, 5).

After the choice, the selected option remained visible for 1 second, then either the certain option was selected and remained visible for another second, or the result of the lottery was displayed for 1 second if the lottery option was selected.

### Quiz task

The quiz task consisted of questions that were drawn from the French version of the ‘Trivial Pursuit’ game. Quiz questions were sorted according to the rate of correct answers given by participants in a previous study and then divided into categories of relatively easy, intermediate, or relatively hard questions.

After a training of 11 trials, the task consisted of 128 trials. In every trial, a quiz question was presented for 3.5 s, followed by 4 possible answers from which one had to be selected within 4.5 s. Hard, easy and intermediate questions were respectively presented during negative episodes, positive episodes and transitions between episodes. The 1 s feedback screen showed a smiling emoji accompanied by a cheerful ping sound (positive feedback) or a frowning emoji accompanied by an unpleasant buzz sound (negative feedback). A player always received positive feedback when answering correctly, and feedback was always negative when the player did not answer within the time limit. Feedback following an incorrect answer depended on a bias parameter whose value depended on the experimental condition: 0%, 25%, or 50% respectively for negative episode, transition, and positive episode. Moreover, questions asked during 50% bias and 0% bias conditions were respectively easier and harder, while during transition questions with intermediate difficulty were used. There was a total of 2 episodes of 50% bias and 2 episodes of 0% bias. Each episode was composed of 7 transition trials, 18 main condition trials and 7 transitions trials. 50% bias and 0% bias conditions always came alternatively but the first one was randomized.

### Riven task

The Riven task is loosely inspired by the Raven matrices used in classic IQ tests such as WISC V [53]. We designed 150 puzzles composed of a series of 3 original stimuli and three possible following items and calibrated them on 60 participants that did not take part in our main experiment (see SI for behavioural statistics on this calibration procedure). Unlike the actual Raven matrices test, each of the three items could logically follow in the series. In the calibration sample, we checked that participants were engaged in the task and did not detect the manipulation. We computed two estimates of perceived difficulty for each stimulus: response coherence across participants and average participant confidence (S1 Appendix). The actual task presented in this paper consists of 75 trials. On each trial, a puzzle was presented for a maximum of 15 seconds, during which the participants had to select the next item in the suite, or answer “none of the above.” Participants were then asked to rate how confident they felt about their answer by selecting a position on a continuous scale presented with the question “How sure am I?,” with anchors “Very sure” and “Not at all sure.” The feedback screen and sound were the same as in the quiz task.

Leveraging on this design that allowed for entirely controlled feedback sequences, we selected two sequences of 75 trials based on average model and parameter recoverability, using the simulation procedure described in the results section (S1 Appendix). At task launch, the order of the puzzles was randomised, while always keeping the 20% easiest (as identified by the calibration procedure) associated with a positive feedback, in order to promote participants’ sense of agency and engagement with the task. Participants were divided into two groups. Participants in the first group were assigned the same sequence for both experimental sessions, while participants in the second group were assigned different sequences in a randomised order.

### Statistical analysis

All the analyses were conducted using MATLAB (Mathworks Inc) version R2025a. Statistical tests were performed using MATLAB native functions and the associated Statistics and Machine Learning toolbox. All the reported p-values are two-tailed.

Because most participants completed two different tasks, the participant groups contributing to each task partially overlapped. For between-task analyses, such as comparisons of computational parameters, we therefore preserved participant identity across tasks rather than treating task-specific samples as independent groups. Specifically, we fitted linear mixed-effects models including task as a categorical fixed effect and participant identity as a random effect, and used these models to derive the relevant omnibus tests and post-hoc contrasts.

Confidence intervals for within-/between-subject variability ratios were estimated by subject-level bootstrap resampling, preserving the pairing between sessions and recomputing the log ratio in each bootstrap sample.

### Modelling

Models were fit to individual participants data, using the VBA academic toolbox in MATLAB [54]. The inversion routine inverts nonlinear state-space models using variational Bayes under the Laplace approximation [55,56]. It also estimates model evidence, representing a trade-off between accuracy (goodness of fit) and complexity (degrees of freedom).Group-level model comparison was performed using random-effects Bayesian model selection, as implemented in the VBA toolbox, using subject-level model evidence estimates as input [38,57]. All the associated Bayesian tests for model frequency distribution differences between groups and conditions were performed using dedicated implementations in VBA.

## Supporting information

S1 Appendix Riven task design procedure. (DOCX)

S2 Appendix Lottery task design details. (DOCX)

S1 Fig Model and parameter recovery for noiseless continuous outcomes. *Left panel*. Confusion matrix (exceedance probability of each model given the model that generated the data). *Middle panel*. Overview of same-parameter recovery for every considered model. Every cell displays the correlation between a generative parameter and the corresponding recovered parameter when the same model is fitted. *Right panel*. Parameter recovery for the selected model. Cross-parameter correlation matrix when the same model is fitted.
(PNG)

S3 Appendix Conditions of identifiability of models with an asymmetry between positive and negative outcomes. (DOCX)

S2 Fig Cross-parameter correlation matrix for every model in the initial model space. X-axis, generative parameter used in the simulation. Y-axis, recovered parameter value after model fit. Values displayed on matrix cells are the Spearman correlation coefficient across 200 simulated subjects. Diagonal cells contain parameter recovery correlations. Models are denoted by their number according to the model space described in the main manuscript.
(PNG)

S3 Fig Effect of the number and frequency of mood ratings on model identifiability for randomly placed ratings. (A) Mean recovery exceedance probability as a function of the proportion of trials in which mood ratings are collected, in two situations: fixed total trial number (red) and fixed total mood rating number (grey pink). Dotted lines denote linear interpolation for missing data. (B) Confusion matrix (exceedance probability of each model given the model that generated the data) for 19 randomly placed mood ratings out of a total of 75 trials, without (top) and with (bottom) linear interpolation for missing data. (C) Confusion matrix for a total of 75 randomly placed mood ratings (300 trials), without (top) and with (bottom) linear interpolation for missing data.
(PNG)

S4 Fig Effect of the number and frequency of mood ratings on parameter recovery for randomly placed ratings. (A) Mean parameter recovery correlation as a function of the proportion of trials in which a mood rating is collected, in two situations: fixed total trial number (blue) and fixed total mood rating number (grey blue). Dashed lines denote linear interpolation for missing data. (B) Overview of same-parameter recovery for every model. Every cell displays the correlation between a generative parameter and the corresponding recovered parameter when the same model is fitted, without (top) and with (bottom) linear interpolation for missing data. (C) Cross-parameter correlation matrices for the selected model, fitted on native only (top) or linearly interpolated (bottom) ratings. Diagonals correspond to the highlighted columns in the middle panel.
(PNG)

S4 Appendix Mood models considering economic choices. (DOCX)

S5 Fig Cross-task parameter stability for tasks completed in a same experimental session. Correlation between the parameters of the selected mood model, that was fit separately to two tasks that were completed in a same experimental session. X-axis denotes parameter estimates in the Riven task. Y-axis denotes parameter estimates in the quiz task (*first row*) or in the lottery task (*second row*). No subject completed both the lottery and quiz tasks. Consistently with the main results, the most stable parameters were baseline mood *ω*_*0*_ and feedback accumulation sensitivity *ω*_*f*_. The correlation is only significant for *ω*_*f*_ values between the Riven and quiz tasks, which may be related to similarities in their designs. The lack of correlation between baseline mood *ω*_*0*_ estimates might relate to the negative time drift frequently observed during this type of tasks, which could dissociate baseline estimates when two tasks are completed sequentially within the same experimental session.
(PNG)

## References

[1] Delay J. *Les Dérèglements de l’humeur*. Presses universitaires de France. 1946.

[2] Ekman P. Basic emotions. Handbook of Cognition and Emotion. 1999.

[3] Ketai R. Affect, mood, emotion, and feeling: semantic considerations. Am J Psychiatry. 1975;132:1215–7. 1166902 10.1176/ajp.132.11.1215

[4] Morris WN, Schnurr PP. Mood: the frame of mind. Springer-Verlag Publishing; 1989.

[5] Kettlewell N, Morris RW, Ho N, Cobb-Clark DA, Cripps S, Glozier N. The differential impact of major life events on cognitive and affective wellbeing. SSM Popul Health. 2019;10:100533. doi: 10.1016/j.ssmph.2019.100533 31909168 PMC6939089

[6] Yarrington JS, Metts AV, Zinbarg RE, Nusslock R, Wolitzky-Taylor K, Hammen CL, et al. The role of positive and negative aspects of life events in depressive and anxiety symptoms. Clin Psychol Sci. 2023;11(5):910–20. doi: 10.1177/21677026221141654 37766940 PMC10530959

[7] Velten J, Lavallee KL, Scholten S, Meyer AH, Zhang X-C, Schneider S, et al. Lifestyle choices and mental health: a representative population survey. BMC Psychol. 2014;2(1):58. doi: 10.1186/s40359-014-0055-y 25628891 PMC4304169

[8] Hatch S, et al. A life course approach to well-being. In: Haworth J, Hart G, editors. Well-being: Individual, community and social perspectives. London: Palgrave Macmillan UK. 2007. p. 187–205. doi: 10.1057/9780230287624_11

[9] Koenders MA, et al. Stressful life events in bipolar I and II disorder: cause or consequence of mood symptoms?. J Affect Disord. 2014;161:55–64. 24751308 10.1016/j.jad.2014.02.036

[10] Otto AR, Eichstaedt JC. Real-world unexpected outcomes predict city-level mood states and risk-taking behavior. PLoS One. 2018;13(11):e0206923. doi: 10.1371/journal.pone.0206923 30485304 PMC6261541

[11] Otto AR, Fleming SM, Glimcher PW. Unexpected but incidental positive outcomes predict real-world gambling. Psychol Sci. 2016;27(3):299–311. doi: 10.1177/0956797615618366 26796614

[12] Westermann R, Spies K, Stahl G, Hesse FW. Relative effectiveness and validity of mood induction procedures: a meta-analysis. Eur J Soc Psychol. 1996;:557–80.

[13] Schulreich S, Heussen YG, Gerhardt H, Mohr PNC, Binkofski FC, Koelsch S, et al. Music-evoked incidental happiness modulates probability weighting during risky lottery choices. Front Psychol. 2014;4:981. doi: 10.3389/fpsyg.2013.00981 24432007 PMC3882660

[14] Eldar E, Rutledge RB, Dolan RJ, Niv Y. Mood as representation of momentum. Trends Cogn Sci. 2016;20(1):15–24. doi: 10.1016/j.tics.2015.07.010 26545853 PMC4703769

[15] Bennett D, Radulescu A, Zorowitz S, Felso V, Niv Y. Affect-congruent attention modulates generalized reward expectations. PLoS Comput Biol. 2023;19(12):e1011707. doi: 10.1371/journal.pcbi.1011707 38127874 PMC10781156

[16] Eldar E, Pessiglione M, Van Dillen L. Positive affect as a computational mechanism. Curr Opin Behav Sci. 2021;39:52–7.

[17] Mason L, Eldar E, Rutledge RB. Mood instability and reward dysregulation-a neurocomputational model of bipolar disorder. JAMA Psychiatry. 2017;74(12):1275–6. doi: 10.1001/jamapsychiatry.2017.3163 29049438

[18] Emanuel A, Eldar E. Emotions as computations. Neurosci Biobehav Rev. 2023;144:104977. doi: 10.1016/j.neubiorev.2022.104977 36435390 PMC9805532

[19] Rutledge RB, Skandali N, Dayan P, Dolan RJ. A computational and neural model of momentary subjective well-being. Proc Natl Acad Sci U S A. 2014;111(33):12252–7. doi: 10.1073/pnas.1407535111 25092308 PMC4143018

[20] Michely J, Eldar E, Martin IM, Dolan RJ. A mechanistic account of serotonin’s impact on mood. Nat Commun. 2020;11(1):2335. doi: 10.1038/s41467-020-16090-2 32393738 PMC7214430

[21] Blain B, Rutledge RB. Momentary subjective well-being depends on learning and not reward. Elife. 2020;9:e57977. doi: 10.7554/eLife.57977 33200989 PMC7755387

[22] Rutledge RB, Skandali N, Dayan P, Dolan RJ. Dopaminergic modulation of decision making and subjective well-being. J Neurosci. 2015;35(27):9811–22. doi: 10.1523/JNEUROSCI.0702-15.2015 26156984 PMC4495239

[23] Eldar E, Niv Y. Interaction between emotional state and learning underlies mood instability. Nat Commun. 2015;6:6149. doi: 10.1038/ncomms7149 25608088 PMC5338993

[24] Vinckier F, Rigoux L, Oudiette D, Pessiglione M. Neuro-computational account of how mood fluctuations arise and affect decision making. Nat Commun. 2018;9(1):1708. doi: 10.1038/s41467-018-03774-z 29700303 PMC5919935

[25] Heerema R, Carrillo P, Daunizeau J, Vinckier F, Pessiglione M. Mood fluctuations shift cost-benefit tradeoffs in economic decisions. Sci Rep. 2023;13(1):18173. doi: 10.1038/s41598-023-45217-w 37875525 PMC10598198

[26] Cecchi R, Vinckier F, Hammer J, Marusic P, Nica A, Rheims S, et al. Intracerebral mechanisms explaining the impact of incidental feedback on mood state and risky choice. Elife. 2022;11:e72440. doi: 10.7554/eLife.72440 35822700 PMC9348847

[27] Gut reactions: a perceptual theory of emotion. https://psycnet.apa.org/record/2007-00936-000

[28] Pessiglione M, Heerema R, Daunizeau J, Vinckier F. Origins and consequences of mood flexibility: a computational perspective. Neurosci Biobehav Rev. 2023;147:105084. doi: 10.1016/j.neubiorev.2023.105084 36764635

[29] Ly V, Wang KS, Bhanji J, Delgado MR. A reward-based framework of perceived control. Front Neurosci. 2019;13:65. doi: 10.3389/fnins.2019.00065 30809112 PMC6379460

[30] Thompson SC. Will it hurt less if i can control it? A complex answer to a simple question. Psychol Bull. 1981;90(1):89–101. doi: 10.1037/0033-2909.90.1.89 7267899

[31] Skinner EA. A guide to constructs of control. J Pers Soc Psychol. 1996;71(3):549–70. doi: 10.1037//0022-3514.71.3.549 8831161

[32] Pessiglione M, Vinckier F, Bouret S, Daunizeau J, Le Bouc R. Why not try harder? Computational approach to motivation deficits in neuro-psychiatric diseases. Brain. 2018;141(3):629–50. doi: 10.1093/brain/awx278 29194534

[33] Pessiglione M, Le Bouc R, Vinckier F. When decisions talk: computational phenotyping of motivation disorders. Curr Opin Behav Sci. 2018;22:50–8.

[34] Montague PR, Dolan RJ, Friston KJ, Dayan P. Computational psychiatry. Trends Cogn Sci. 2012;16(1):72–80. doi: 10.1016/j.tics.2011.11.018 22177032 PMC3556822

[35] Stephan KE, Mathys C. Computational approaches to psychiatry. Curr Opin Neurobiol. 2014;25:85–92. doi: 10.1016/j.conb.2013.12.007 24709605

[36] Palminteri S, Wyart V, Koechlin E. The Importance of Falsification in Computational Cognitive Modeling. Trends Cogn Sci. 2017;21(6):425–33. doi: 10.1016/j.tics.2017.03.011 28476348

[37] Jangraw DC, Keren H, Sun H, Bedder RL, Rutledge RB, Pereira F, et al. A highly replicable decline in mood during rest and simple tasks. Nat Hum Behav. 2023;7(4):596–610. doi: 10.1038/s41562-023-01519-7 36849591 PMC10192073

[38] Rigoux L, Stephan KE, Friston KJ, Daunizeau J. Bayesian model selection for group studies - revisited. Neuroimage. 2014;84:971–85. doi: 10.1016/j.neuroimage.2013.08.065 24018303

[39] Ni Y, Sun J, Li J. The shadowing effect of initial expectation on learning asymmetry. PLoS Comput Biol. 2023;19(7):e1010751. doi: 10.1371/journal.pcbi.1010751 37486955 PMC10399892

[40] Rosenbaum GM, Grassie HL, Hartley CA. Valence biases in reinforcement learning shift across adolescence and modulate subsequent memory. Elife. 2022;11:e64620. doi: 10.7554/eLife.64620 35072624 PMC8786311

[41] Ciranka S, Linde-Domingo J, Padezhki I, Wicharz C, Wu CM, Spitzer B. Asymmetric reinforcement learning facilitates human inference of transitive relations. Nat Hum Behav. 2022;6(4):555–64. doi: 10.1038/s41562-021-01263-w 35102348 PMC9038534

[42] Karvelis P, Paulus MP, Diaconescu AO. Individual differences in computational psychiatry: a review of current challenges. Neurosci Biobehav Rev. 2023;148:105137. doi: 10.1016/j.neubiorev.2023.105137 36940888

[43] Rutledge RB, Moutoussis M, Smittenaar P, Zeidman P, Taylor T, Hrynkiewicz L, et al. Association of neural and emotional impacts of reward prediction errors with major depression. JAMA Psychiatry. 2017;74(8):790–7. doi: 10.1001/jamapsychiatry.2017.1713 28678984 PMC5710549

[44] Vanhasbroeck N, Devos L, Pessers S, Kuppens P, Vanpaemel W, Moors A, et al. Testing a computational model of subjective well-being: a preregistered replication of Rutledge *et al*. (2014). Cogn Emot. 2021;35(4):822–35. doi: 10.1080/02699931.2021.1891863 33632071

[45] Rutledge RB, de Berker AO, Espenhahn S, Dayan P, Dolan RJ. The social contingency of momentary subjective well-being. Nat Commun. 2016;7:11825. doi: 10.1038/ncomms11825 27293212 PMC4909984

[46] Will G-J, Rutledge RB, Moutoussis M, Dolan RJ. Neural and computational processes underlying dynamic changes in self-esteem. Elife. 2017;6:e28098. doi: 10.7554/eLife.28098 29061228 PMC5655144

[47] Chew B, Blain B, Dolan RJ, Rutledge RB. A neurocomputational model for intrinsic reward. J Neurosci. 2021;41(43):8963–71. doi: 10.1523/JNEUROSCI.0858-20.2021 34544831 PMC8549542

[48] Keren H, Zheng C, Jangraw DC, Chang K, Vitale A, Rutledge RB, et al. The temporal representation of experience in subjective mood. Elife. 2021;10:e62051. doi: 10.7554/eLife.62051 34128464 PMC8241441

[49] American psychiatric association. Diagnostic and statistical manual of mental disorders: DSM-5. Washington: 2013.

[50] Brainard DH. The psychophysics toolbox. Spat Vis. 1997;10(4):433–6. doi: 10.1163/156856897x00357 9176952

[51] The VideoToolbox software for visual psychophysics: transforming numbers into movies. PubMed. 1997. 9176953

[52] Kleiner M, et al. What’s new in psychtoolbox-3. Perception. 2007;36:1–16.

[53] Gienger CA. Wechsler intelligence scale for children – Fifth Edition (WISC-V). Lern Lernstörungen. 2018;7:121–4.

[54] Daunizeau J, Adam V, Rigoux L. VBA: a probabilistic treatment of nonlinear models for neurobiological and behavioural data. PLoS Comput Biol. 2014;10(1):e1003441. doi: 10.1371/journal.pcbi.1003441 24465198 PMC3900378

[55] Friston K, Mattout J, Trujillo-Barreto N, Ashburner J, Penny W. Variational free energy and the Laplace approximation. Neuroimage. 2007;34(1):220–34. doi: 10.1016/j.neuroimage.2006.08.035 17055746

[56] Daunizeau J, Friston KJ, Kiebel SJ. Variational bayesian identification and prediction of stochastic nonlinear dynamic causal models. Physica D. 2009;238(21):2089–118. doi: 10.1016/j.physd.2009.08.002 19862351 PMC2767160

[57] Stephan KE, Penny WD, Daunizeau J, Moran RJ, Friston KJ. Bayesian model selection for group studies. Neuroimage. 2009;46(4):1004–17. doi: 10.1016/j.neuroimage.2009.03.025 19306932 PMC2703732

