# Supplementary material for: Assessing the validity and reliability of computational phenotyping of mood
Source: PLoS Comput Biol. 2026 Aug 3;22(8):e1014597. doi: 10.1371/journal.pcbi.1014597 (PMC13456276; doi:10.1371/journal.pcbi.1014597)
Supplement: Supplementary file 1 [file pcbi.1014597.s001.docx]

### Assessing the validity and reliability of computational phenotyping of mood

Pablo Carrillo*, Marc Benhamou*, Roeland Heerema, Jean Daunizeau, Mathias Pessiglione & Fabien Vinckier

S1 Appendix: Riven task design procedure

The Riven task is loosely inspired by Raven’s Progressive Matrices, which are commonly used in classical IQ assessments such as the WISC-V. For the purpose of this task, we developed 150 puzzles, each consisting of a series of three original stimuli and three possible subsequent items. The puzzles were divided into distinct domains intended to engage different cognitive processes and/or stimulus types (e.g., numbers, words, abstract shapes). Examples are provided below. The percentages shown at the bottom of each item represent the proportion of participants who selected that item as their response.

**Riven task design: puzzle examples.** Each puzzle consisted of a series of three stimuli and three possible subsequent items. Percentages below each item indicate the proportion of participants who selected that item during the calibration phase.

In a first calibration step, all these puzzles were presented to 60 healthy participants (these participants did not participate in our main experiment). The design of the calibration was similar to that of the Riven task except that:

- the response time limit was increased to 60 s;
- there was no “None of the above” response option;
- no feedback was provided;
- there were no economic choices or mood ratings.

**Riven task calibration procedure: trial overview.** Each trial consisted of only two screens, with a puzzle question and a confidence rating.

In this initial group of participants, the mean response time was 15 seconds (SD = 4.6), and the mean confidence rating was 55.7%. Notably, we observed substantial variability across puzzles in what we term response homogeneity, defined as the difference between the most and least frequently chosen options.

We also conducted basic validity checks. As expected, puzzles associated with higher confidence ratings were solved more quickly than those with lower average confidence (Pearson’s *r* = –0.78, *p* < .001). Furthermore, within a given puzzle, the option selected by the majority of participants was endorsed with higher confidence than the option chosen by the minority (*t* = 4.9, *df* = 141, *p* < .001).

**Riven task calibration results.** *Left:* scatter plot of per stimulus reaction time and confidence. (*** *p* < .001). *Right:* Distribution of response homogeneity across stimuli. Response homogeneity is computed as the difference in proportion between the most chosen and the least chosen option.

Importantly, in subsequent experimental sessions where feedbacks were included, none of the participants detected that they were uncorrelated to their choices. Specifically, all participants completed a post-task funnel questionnaire, and none reported that the task was misleading, that the feedback was inconsistent with their responses, or that the feedback was random.

Having validated the possibility of using entirely pre-determined outcome, we then designed binary feedback sequences meant to maximise statistical power to separate the computational models of mood considered in our model space. To this end, we first generated 300 sequences, with random binary outcomes following a constrained structure. Each sequence was composed of alternating mini-blocks of higher or lower positive feedback rate that were intended to introduce temporal structure, with a maximum of 10 blocks. Mini-blocks varied in length from 5 to 15 trials (drawn with equal probability) and in dominant feedback rate, with values of .55, .65 or .75 (also drawn with equal probability). Positive-dominant and negative-dominant blocks always alternated. Feedback values were then drawn from binomial distributions, using the proportions specific to every block.

For each of these sequences, mood ratings were simulated for 200 synthetic subjects, using the procedure described in the main manuscript results section. All 10 mood models included in the model space were fit to subject data. In line with the simulation-recovery procedure, we then used random-effects Bayesian model selection to compute exceedance probabilities for each model and generate confusion matrices. We also computed the correlation between generative and fitted parameters to generate parameter recovery matrices. We then computed the traces of these matrices, which summarise model and parameter recoverability, for each sequence, and selected the two sequences with the highest traces.

**Group average mood rating as a function of feedback sequence.** Each sequence of 75 binary feedbacks is depicted as red dots (positive or negative). The time course of average z-scored mood is also shown, with the shaded area representing the standard error of the mean.
