# Supplementary material for: Assessing the validity and reliability of computational phenotyping of mood
Source: PLoS Comput Biol. 2026 Aug 3;22(8):e1014597. doi: 10.1371/journal.pcbi.1014597 (PMC13456276; doi:10.1371/journal.pcbi.1014597)
Supplement: Supplementary file 2 [file pcbi.1014597.s002.docx]

### Assessing the validity and reliability of computational phenotyping of mood

Pablo Carrillo*, Marc Benhamou*, Roeland Heerema, Jean Daunizeau, Mathias Pessiglione & Fabien Vinckier

### S2 Appendix: Lottery task design details

After 10 training trials designed to familiarize participants with the task and each question, participants completed 150 trials. The task included three trial types: 50 gain trials, 50 loss trials, and 50 mixed trials. In gain trials, participants chose between a certain gain and an equal-probability lottery between 0€ and a larger gain. In loss trials, they chose between a certain loss and an equal-probability lottery between 0€ and a larger loss. In mixed trials, they chose between a certain amount of 0€ and an equal-probability lottery between a gain and a loss amount.

At the beginning of each trial, a fixation cross was displayed for 1 s. The two options were then presented on the left and right sides of the screen. The side on which the lottery option appeared alternated every 10 trials. Participants had 10 s to make their choice. If they failed to respond within this time limit, the worst possible outcome for that trial was selected. After a response, the unchosen option disappeared from the screen and the selected option remained visible for 1 s. If the certain option had been selected, the certain outcome was then displayed for 1 s. If the lottery option had been selected, the lottery outcome was revealed and displayed for 1 s.

The certain option ranged from −0.60€ to 0€ in loss trials, from 0€ to 0.60€ in gain trials, and was fixed at 0€ in mixed trials. The lottery option involved a gain between 0€ and 3€ or a loss between 0€ and −3€. More specifically, in mixed trials, there were five possible gain amounts, in euro cents: 30, 50, 80, 110, and 150. The corresponding loss amount was determined by applying one of ten multipliers to the gain amount: 0.2, 0.3, 0.4, 0.52, 0.66, 0.82, 1, 1.2, 1.5, or 2. In gain trials, there were five certain amounts: 20, 30, 40, 50, and 60 cents. Lottery gains were determined by applying one of ten multipliers to the certain amount: 1.68, 1.82, 2, 2.22, 2.48, 2.8, 3.16, 3.6, 4.2, or 5. In loss trials, the same five certain amounts were used with negative values: −20, −30, −40, −50, and −60 cents, and lottery losses were determined using the same ten multipliers. Trial order was randomized across the 150 trials.

Each trial ended with a mood rating. Participants were asked “Comment je me sens ?” and rated their current mood by placing a cursor on a continuous scale ranging from “De mauvaise humeur” to “De bonne humeur.” At screen onset, there was no cursor on the scale; participants responded by touching a tactile screen, meaning there was no obvious difference in motor cost between segments of the rating scale. Participants had up to 5 s to provide their rating. If they responded before the end of this period, their selected rating remained visible for the remainder of the 5-s rating period. If no response was provided within 5 s, no mood value was recorded for that trial and participants were prompted to respond faster.

The task was adapted from Rutledge et al. (2014), with several differences. First, participants had 10 s to make their choice, whereas Rutledge et al. used a 3-s choice time limit. Second, the feedback timing differed: in the present task, the selected option remained visible for 1 s and the outcome was then displayed for 1 s, whereas in Rutledge et al., certain choices remained on the screen for 7 s and lottery choices remained on the screen for 6 s before the outcome was revealed for 1 s. Third, the present task used a fixed 1-s fixation period before each trial; Rutledge et al. used a 3–11 s jittered intertrial interval with a saturated Gamma distribution in their fMRI experiment design, no specific description was specified for behaviour-only experiments. Fourth, mood ratings were collected after every trial, whereas Rutledge et al. collected happiness ratings after every two to three trials. Finally, the wording of the affective rating differed: participants in the present study rated their mood on a scale from “bad mood” to “good mood,” whereas Rutledge et al. asked participants how happy they were at that moment on a scale from “very unhappy” to “very happy.”
