# Supplementary material for: Assessing the validity and reliability of computational phenotyping of mood
Source: PLoS Comput Biol. 2026 Aug 3;22(8):e1014597. doi: 10.1371/journal.pcbi.1014597 (PMC13456276; doi:10.1371/journal.pcbi.1014597)
Supplement: Supplementary file 3 [file pcbi.1014597.s004.docx]

### Assessing the validity and reliability of computational phenotyping of mood

Pablo Carrillo*, Marc Benhamou*, Roeland Heerema, Jean Daunizeau, Mathias Pessiglione & Fabien Vinckier

### S3 Appendix: conditions of identifiability of models with an asymmetry between positive and negative outcomes

The model simulation results showed that no model including an asymmetry between positive and negative outcomes, scaled by parameter *a*, was recoverable. This result is surprising, given that (i) this type of specification is frequently used in the field of reinforcement learning, where a positivity bias (*a* > 0) has consistently been reported (Palminteri & Lebreton, 2022) and (ii) analytically, the asymmetry parameter *a* can be distinguished from the other model parameters when the outcome sequence is designed for this purpose.

Using our model simulation results, we studied the influence of model specification and parameter values on the identifiability of models that included *a.* To do so, we ranked our simulated subjects along the factor dimension of interest and compared recovery across the different models including *a*. Specifically, for each of these models, we defined identifiability as selection of the model by random-effects Bayesian model comparison, relative to its direct counterpart without the asymmetry effect. We used this restricted model space for model comparison because the closest model that didn’t include an asymmetry term had been systematically selected in our initial analysis.

We found that the primary factors determining model identifiability were (i) the strength of the asymmetry bias, defined as the distance of parameter *a* from neutral value 1, |*a*-1| and (ii) the absolute asymmetry deviation, defined as the strength of the bias multiplied by |*ω_f_*|. Comparing models M7, M8, M9 and M10 two by two, we also found that other model specifications influenced model recovery:

- Adding an EV accumulation term increased the minimum asymmetry-bias, or absolute-asymmetry-deviation, threshold required for model recovery.
- Adding a reciprocal effect of mood, scaled by parameter δ, further increased this threshold and generated intervals of non-identifiability above the threshold, producing non-monotonic patterns that suggest more complex interactions.

We also noted that, in noisy simulations, models with asymmetry became partially identifiable (main manuscript, Fig. 3). Moreover, in simulations with continuous outcomes, the asymmetry parameter recovery correlations seemed to increase as compared to binary outcomes (S1 Fig.; main manuscript, Fig. 2).

In conclusion, models that included an asymmetry between negative and positive outcomes were not intrinsically unrecoverable (i.e., not overparametrized). Rather, the lack of identifiability that we observed appears to result from the interaction of several factors, including generative parameter values, model specifications, binary outcomes and noise level.

Palminteri, S., & Lebreton, M. (2022). The computational roots of positivity and confirmation biases in reinforcement learning. *Trends in Cognitive Sciences*, *26*(7), 607–621. https://doi.org/10.1016/j.tics.2022.04.005
