# Supplementary material for: Assessing the validity and reliability of computational phenotyping of mood
Source: PLoS Comput Biol. 2026 Aug 3;22(8):e1014597. doi: 10.1371/journal.pcbi.1014597 (PMC13456276; doi:10.1371/journal.pcbi.1014597)
Supplement: Supplementary file 4 [file pcbi.1014597.s008.docx]

### Assessing the validity and reliability of computational phenotyping of mood

Pablo Carrillo*, Marc Benhamou*, Roeland Heerema, Jean Daunizeau, Mathias Pessiglione & Fabien Vinckier

### S4 Appendix: Mood models considering economic choices

In order to fit mood models that include value for the economic choices, choice behaviour was first analysed using a computational discounting model fitted separately at the participant and session levels. On each trial, participants chose between an uncostly option and a costly option, where the cost could correspond to delay, risk, physical effort, or mental effort. The subjective value of the uncostly option was defined as *V_uncostly_ = k_rew_R_uncostly_*, where *R* denotes reward magnitude and *k_rew_* captures reward sensitivity. Building on previous literature (Heerema et al., 2023), subjective value of the costly option depended on the choice type:

$$\begin{matrix} V_{delay}=k_{rew}R_{costly}e^{-k_{D}D} \\ V_{risk}=k_{rew}R_{costly}\left( 1-p_{L} \right)-k_{R}p_{L}L \\ \begin{matrix} V_{physical}=k_{rew}R_{costly}-k_{PE}{PE}^{2} \\ V_{mental}=k_{rew}R_{costly}-k_{ME}{ME}^{2} \end{matrix} \end{matrix}$$

where *D* denotes delay, *p_L_* denotes the probability of a loss, *L* denotes loss magnitude, and *PE* and *ME* denote physical and mental effort levels, respectively. Choice probability was then modelled as a logistic function of the difference between the costly and uncostly option values, *ΔV=V_costly_−V_uncostly_*, with choice-type-specific inverse-temperature and bias parameters:

$$P\left( costly \right)= \frac{1}{1+e^{-\left( \beta_{c}\Delta V+b_{c} \right)}}$$

where *c* indexes the choice type. Model parameters therefore captured reward sensitivity, cost sensitivity for each domain, and choice stochasticity/bias for each choice type.

After fitting this model, we retrieved for each subject and trial the value of the chosen option, in order to build an economic-value accumulation term that could be additively included in mood computational models. Specifically, we included the two following models in our model comparison space:

- Eco1:	$m_{(t)}={\omega_{0}+ \omega}_{f}\sum_{j=1}^{t} \gamma^{t-j}f_{\left( j \right)}+\omega_{eco}\sum_{j=1}^{t} \gamma^{t-j}V_{(j)}+ \omega_{t}t$
- Eco2:	$\begin{matrix} m_{(t)}={\omega_{0}+ \omega}_{f}\sum_{j=1}^{t} \gamma^{t-j}f_{(j)}+\omega_{eco}\sum_{j=1}^{t} \gamma^{t-j}V_{(j)}+ \omega_{t}t \\ f_{\left( t \right)}={out}_{\left( t \right)}+\delta\cdot m_{(t-1)} \end{matrix}$

where *V_(j)_* denotes the value of the chosen option at trial *j*, *ω_eco_* is a free parameter that represents the weight of economic value accumulation (relative to feedback and time drift), and the other notations are the same as in the main manuscript. Relative to the model space described in the main manuscript, Eco2 has the same specifications as the selected model M5, except for the added economic value term. Eco1 is a simpler variant, based on model M3 (the simplest mood model with outcome accumulation), to which it adds the economic value term. The whole extended model space was thereby composed of 8 models.

Following the same procedure as in the main analysis, we first tested for difference between task-groups, and found strong evidence that model frequency distribution did not differ between the task-groups (xp > .99 for both sessions). Within each task-group, model frequency distribution did not differ between sessions (xp > .99 for every task-group). We then pooled all subjects together and performed random-effects Bayesian model comparison using only the first session. The selected model was M5, the same model as in the main analysis that considered only 6 models (xp > .99). We concluded that economic choice was not a key driver of mood fluctuations in these tasks.

**References**

Heerema, R., Carrillo, P., Daunizeau, J., Vinckier, F., & Pessiglione, M. (2023). Mood fluctuations shift cost-benefit tradeoffs in economic decisions. *Scientific Reports*, *13*(1), 18173. https://doi.org/10.1038/s41598-023-45217-w
