# Supplementary material for: Assessing the validity and reliability of computational phenotyping of mood
Source: PLoS Comput Biol. 2026 Aug 3;22(8):e1014597. doi: 10.1371/journal.pcbi.1014597 (PMC13456276; doi:10.1371/journal.pcbi.1014597)
Supplement: Supplementary file 5 [file pcbi.1014597.s011.docx]

*Reviewer #1: The manuscript presents a useful characterization of common models of mood in terms of identifiability, and an empirical test of the stability of their parameters in the context of three mood manipulation tasks. The effects of outcomes on mood are found to be stable and replicable across tasks. Other aspects such as the effect of mood on subjective outcome, and the asymmetry in mood updates, were less identifiable. The work makes a meaningful contribution to the literature on computational models of mood, but the following issues need to be addressed:*

We thank the reviewer for their positive assessment of our work and for their constructive comments.

*It needs to be acknowledged that the choice of tasks may have a lot to do with the observed results. The tasks represent a particular approach to manipulating mood, which uses relatively small recurring outcomes and does not engage learning (learning likely happens in some ways but it is not quantified or controlled). This approach is popular, and this makes the authors' choice reasonable, but the results have to be interpreted in this light because (1) effects of mood on subjective outcome have not only but primarily been demonstrated when mood was manipulated using big singular outcomes, and (2) by some accounts mood is driven by learning from reward prediction errors, rather than by the reward prediction errors themselves. A different task might therefore have led to more stable and identifiable effects of mood on subjective outcome, and to a more identifiable role for EV in shaping mood.*

This is a relevant point to better integrate the present manuscript into the existing literature. We added the following paragraph in the main text discussion (lines 479-493), to acknowledge this limitation and discuss how it relates to previous findings:

*At a more general level, all the tasks used in this study consider mood fluctuations in a stable environment, in the absence of explicit learning: the outcomes experienced by participants may have motivational value, but they do not carry relevant information to learn about the task. While this approach is the most frequently used in the literature, one line of work focuses on interactions between learning-relevant variables and mood^21,23^. In particular, the reciprocal effect of mood on feedback perception has primarily been found when big singular outcomes were added to a task, arguably leveraging a learning mechanism^23^; it was conceptualised to be relevant in a context where mood interacts primarily with learning^14^. Using tasks where learning about outcomes is a key dimension might therefore lead to different findings. One can speculate that when made relevant by active learning, associated variables such as expected values and RPEs might carry more relative weight in driving mood fluctuations, potentially leading to the selection of a different model. Likewise, different models that consider an effect of mood on subjective outcome in a learning context might yield different results regarding the recoverability and stability of the associated parameters. More generally, these different empirical approaches relate to different conceptualisations of the function of mood as driving primarily reinforcement learning or motivation.^14,28^*

*The unidentifiability of an asymmetry in mood updates is currently not convincingly explained. In theory, when all outcomes equal zero, then only w_0 can have an effect on predicted mood, so this parameter should be dissociable from both w_f and alpha. Furthermore, when all outcomes are negative, then only w_0 and w_f can influence mood and not alpha. Together, these two dissociations should enable identifying the three parameters. Likewise, the results of the parameter recovery test do not seem to support the lack of dissociation proposed by the authors, as the spurious correlations between alpha and w_0/w_f are fairly low.*

We want to thank the reviewer for raising this point. Indeed, the explanation we suggested for this lack of identifiability in the first version of the manuscript was incorrect.

Regarding the more specific remarks, we agree that the low correlation between w_0/w_f and alpha does not support the initial explanations. We also agree with the analytical point put forward by the reviewer. In order to clarify the conditions that lead to models including the asymmetry parameter to become unidentifiable, we conducted specific analyses that we reported in a dedicated SI file (S3 Appendix):

*The model simulation results showed that no model including an asymmetry between positive and negative outcomes, scaled by parameter a, was recoverable. This result is surprising, given that (i) this type of specification is frequently used in the field of reinforcement learning, where a positivity bias (a > 0) has consistently been reported (Palminteri & Lebreton, 2022) and (ii) analytically, the asymmetry parameter a can be distinguished from the other model parameters when the outcome sequence is designed for this purpose.*

*Using our model simulation results, we studied the influence of model specification and parameter values on the identifiability of models that included a. To do so, we ranked our simulated subjects along the factor dimension of interest and compared recovery across the different models including a. Specifically, for each of these models, we defined identifiability as selection of the model by random-effects Bayesian model comparison, relative to its direct counterpart without the asymmetry effect. We used this restricted model space for model comparison because the closest model that didn’t include an asymmetry term had been systematically selected in our initial analysis.*

*We found that the primary factors determining model identifiability were (i) the strength of the asymmetry bias, defined as the distance of parameter a from neutral value 1, |a-1| and (ii) the absolute asymmetry deviation, defined as the strength of the bias multiplied by |ω_f_|. Comparing models M7, M8, M9 and M10 two by two, we also found that other model specifications influenced model recovery:*

- *Adding an EV accumulation term increased the minimum asymmetry-bias, or absolute-asymmetry-deviation, threshold required for model recovery.*
- *Adding a reciprocal effect of mood, scaled by parameter δ, further increased this threshold and generated intervals of non-identifiability above the threshold, producing non-monotonic patterns that suggest more complex interactions.*

*We also noted that, in noisy simulations, models with asymmetry became partially identifiable (main manuscript, figure 3). Moreover, in our simulations with continuous outcomes, the asymmetry parameter recovery correlations seemed to increase as compared to binary outcomes (S1 Fig.; main manuscript, Fig. 2).*

*In conclusion, models that included an asymmetry between negative and positive outcomes were not intrinsically unrecoverable. Rather, the lack of identifiability that we observed appears to result from the interaction of several factors, including generative parameter values, model specifications, binary outcomes and noise level.*

*Presumably the surprising results regarding interpolation reflect the unsuitability of the linear functional form of the interpolation. It could be worth while to test other forms such as Gaussian filtering or spline interpolation.*

Although this type of interpolation has not been used in the literature, we agree that this methodological article was a good opportunity to test different interpolation methods. Specifically, we conducted the same analyses previously done with linear interpolation, using the following methods:

- Spline interpolation, as implemented in MATLAB’s “fillmissing” function
- Gaussian-kernel regression, as implemented with MATLAB’s “fitrkernel” function, with a fixed Gaussian kernel of *σ = n*, where *n* denotes the downsampling rate.

Both methods yielded the same results as the linear interpolation we initially used. We reproduce below analogous figures to the ones shown in the original manuscript with linear interpolation.

Spline interpolation:

Gaussian-kernel regression:

*Line 169: was some specific noise distribution assumed around the predicted m?*

The previous version of the manuscript did not include any noisy simulation. We agree that it considerably restricted the scope of the findings. In the revised manuscript, we included a new section dedicated to the analysis of the effect of noise on model and parameter recovery, with a focus on realistic noise level estimated from the empirical dataset (lines 230-260 and Fig. 3):

*In order to assess the effect of noisy mood ratings, we incorporated a reliability parameter α controlling the relative contribution of the model-predicted signal and random response variability. Specifically, after mapping the latent model prediction onto the bounded response scale, the observed response was generated as a mixture of this bounded signal and a random contaminating component:*

$$y_{t}=\alpha m_{r(t)}+\left( 1-\alpha\right)\epsilon_{t}$$

*where ϵ_t_ was sampled from a uniform distribution over the same interval, ϵ_t_ ∼ U (−1,1), and α∈ [0,1]. This formulation ensures that simulated observations remain within the admissible response range for all values of α. The parameter α can be interpreted as a reliability or signal-preservation parameter: when α = 1, responses are fully determined by the model-predicted signal, whereas when α = 0, responses are independent of the model and uniformly distributed over the response scale. Intermediate values correspond to partial corruption of the model-predicted signal by bounded random variability.*

*Systematically assessing model and parameter recovery with varying α values, we found that global model identifiability plateaus until α values around 0.5 (corresponding to a signal-to-noise ratio of 1), before decreasing sharply. By contrast, parameter recovery follows a seemingly linear decrease until α values around 0.3 before collapsing (Fig 3A and 3D).*

*To assess the global reliability of mood ratings in the experimental dataset, we estimated the empirically predictable variance using a global mixed-effects linear model that included lagged Volterra predictors and autoregressive terms for past mood ratings. This analysis yielded a R2 of 0.865, corresponding to an estimated reliability parameter α = 0.717, with the remaining unexplained variance taken as a plausible estimate of empirical noise.*

*Examining confusion matrices around this value, we found that the model recovery pattern remained close to the noiseless reference, with perfect identification of models that didn’t include an asymmetry parameter (all xp > .99, Fig. 3A-C). Consistent with the global pattern, parameter recovery was more impacted by noise than model identifiability at the empirical estimate. Recovery remained high ω for parameters (r > 0.67, 95%CI lower bound > .59), but fell to intermediate levels for the decay factor γ (0.51 < r < 0.94), which became sensitive to the presence of the parameter that governs the reciprocal effort of mood on feedback perception, δ (Fig. 3D-F). The two remaining parameters a and δ showed low recoverability (r < .40, 95%CI higher bound < .50).*

**Figure 3. Effect of mood rating reliability on model and parameter recovery.** (A) Mean model recovery exceedance probability as a function of decreasing signal weight. (B) Confusion matrix (exceedance probability of each model given the model that generated the data) of the full model space for the experimental noise estimate (α = 0.72). (C) Confusion matrix of the restricted model space for the experimental noise estimate. (D) Mean parameter recovery correlation as a function of decreasing signal weight. (E) Same-parameter recovery across models for the experimental noise estimate (α = 0.72). (F) Selected model cross-parameter correlation for the experimental noise estimate.

*Reviewer #2: This study investigates computational models of mood dynamics during decision-making, and introduces the novel Riven task designed to control feedback sequences while preserving participants' sense of agency over outcomes. The work addresses three key objectives: (1) examining how feedback sequences influence the free parameters governing short-term mood fluctuations, (2) assessing the robustness of existing mood models in terms of model identification and parameter recovery, and (3) evaluating test-retest consistency by repeating the task after a two-week interval. The authors demonstrate that a representative set of mood dynamics models - previously employed in the literature - exhibit robust validity and satisfactory test-retest reliability, though some models proved non-identifiable. Building on the authors' previous work (Vinckier et al., 2018; Heerema et al., 2023; Pessiglione et al., 2023), this study makes a valuable contribution to the literature on mood dynamics. While the manuscript is well-structured, addressing the following points could further enhance its clarity and rigour:*

We are grateful to the reviewer for their positive evaluation of our manuscript and for their relevant comments.

*1. The economic choice task, though not the primary focus, may exert unintended influence on mood verbal reports. Even in the absence of explicit feedback, performing the task could evoke implicit expectations about future gains or losses. These expectations, in turn, may independently modulate participants’ mood states, adding an additional layer of complexity to the estimation of the expected value (EV), beyond the challenges already raised in the discussion section (lines 537–552). To what extent might this unintended influence bias key findings, such as the selection of model 5 over models including the EV term?*

This is an important point, and we agree it was not clearly addressed in the original manuscript. We reported this analysis and results in a dedicated SI file (S4 Appendix):

*In order to fit mood models that include value for the economic choices, choice behaviour was first analysed using a computational discounting model fitted separately at the participant and session levels. On each trial, participants chose between an uncostly option and a costly option, where the cost could correspond to delay, risk, physical effort, or mental effort. The subjective value of the uncostly option was defined as V_uncostly_ = k_rew_R_uncostly_, where R denotes reward magnitude and k_rew_ captures reward sensitivity. Building on previous literature (Heerema et al., 2023), subjective value of the costly option depended on the choice type:*

$$\begin{matrix} V_{delay}=k_{rew}R_{costly}e^{-k_{D}D} \\ V_{risk}=k_{rew}R_{costly}\left( 1-p_{L} \right)-k_{R}p_{L}L \\ \begin{matrix} V_{physical}=k_{rew}R_{costly}-k_{PE}{PE}^{2} \\ V_{mental}=k_{rew}R_{costly}-k_{ME}{ME}^{2} \end{matrix} \end{matrix}$$

*where D denotes delay, p_L_ denotes the probability of a loss, L denotes loss magnitude, and PE and ME denote physical and mental effort levels, respectively. Choice probability was then modelled as a logistic function of the difference between the costly and uncostly option values, ΔV=V_costly_−V_uncostly_, with choice-type-specific inverse-temperature and bias parameters:*

$$P\left( costly \right)= \frac{1}{1+e^{-\left( \beta_{c}\Delta V+b_{c} \right)}}$$

*where c indexes the choice type. Model parameters therefore captured reward sensitivity, cost sensitivity for each domain, and choice stochasticity/bias for each choice type.*

*After fitting this model, we retrieved for each subject and trial the value of the chosen option, in order to build an economic-value accumulation term that could be additively included in mood computational models. Specifically, we included the two following models in our model comparison space:*

- *Eco1:*	$m_{(t)}={\omega_{0}+ \omega}_{f}\sum_{j=1}^{t} \gamma^{t-j}f_{\left( j \right)}+\omega_{eco}\sum_{j=1}^{t} \gamma^{t-j}V_{(j)}+ \omega_{t}t$
- *Eco2:*	$\begin{matrix} m_{(t)}={\omega_{0}+ \omega}_{f}\sum_{j=1}^{t} \gamma^{t-j}f_{(j)}+\omega_{eco}\sum_{j=1}^{t} \gamma^{t-j}V_{(j)}+ \omega_{t}t \\ f_{\left( t \right)}=u_{\left( t \right)}+\delta\cdot m_{(t-1)} \end{matrix}$

*where V_(j)_ denotes the value of the chosen option at trial j, ω_eco_ is a free parameter that represents the weight of economic value accumulation (relative to feedback and time drift), and the other notations are the same as in the main manuscript. Relative to the model space described in the main manuscript, Eco2 has the same specifications as the selected model M5, except for the added economic value term. Eco1 is a simpler variant, based on model M3 (the simplest mood model with outcome accumulation), to which it adds the economic value term. The whole extended model space was thereby composed of 8 models.*

*Following the same procedure as in the main analysis, we first tested for difference between task-groups, and found strong evidence that model frequency distribution did not differ between the task-groups (xp > .99 for both sessions). Within each task-group, model frequency distribution did not differ between sessions (xp > .99 for every task-group). We then pooled all subjects together and performed random-effects Bayesian model comparison using only the first session. The selected model was M5, the same model as in the main analysis that considered only 6 models (xp > .99). We concluded that economic choice was not a key driver of mood fluctuations in these tasks.*

*2. The finding that EV does not influence mood in the tested models is surprising, particularly in light of the well-documented link between Reward Prediction Error (RPE) and affective valence. The authors acknowledge that EV estimation may be challenging in the quiz and Riven tasks, but less so in the gambling task. The authors could supplement the model-free analyses with an examination of RPE (= feedback – EV) effects on mood. This could be visualised in Figure 4, providing a more comprehensive understanding of the relationship between RPE and mood fluctuations, at least within the gambling task.*

We entirely agree with the reviewer that this failure to replicate the finding that RPE is the primary driver of mood fluctuations in the lottery task is intriguing. Following their suggestion, we updated the model-free results figure with the effect of RPE on mood. We also decided to describe the relation between RPE and mood ratings in the model-free results section, in parallel to the relation between outcome and mood ratings (lines 321-326 and Fig. 6):

*Substituting RPE to reward yielded the same result, albeit with lower correlation coefficients: there were significant correlations between RPE and mood ratings in the lottery (r = 0.063, 95%CI [.026, 1.0]), quiz (r = 0.14, 95%CI [.11, .18]), and Riven task (r = 0.16, 95%CI [.13, .20]); this correlation was lower in the lottery task than in the quiz (t(316) = 3.63, p < .001) and Riven task (t(382) = 3.92, p < .001), while there was no difference between these last two tasks (t(484) = .95, p = .34).*

*3. The asymmetry term appears to be closely related to loss aversion, a widely recognised cognitive bias in economic and psychological models. Could the identifiability of the models including an asymmetry term be improved by employing mixed lotteries (combining gains and losses) instead of the pure gain/loss lotteries used in this study?*

We agree with the reviewer that this finding is surprising, especially given the considerable behavioural economic literature that consistently reported a positivity bias using this kind of parametrization. However, mood models differ slightly from value learning models typically used in this kind of analyses.

This being said, we agree that our previous explanation for the non-identifiability of these models was not convincing. Following the suggestions of reviewer 1, we conducted supplementary analyses to precisely assess the conditions of identifiability of these models. These analyses are reported in a dedicated SI file (S3 Appendix). In brief, we found that models that included an asymmetry between negative and positive outcomes were not overparametrized; rather, they were difficult to recover due to an interaction between the range of generative parameter values, model specifications, binarity of outcomes and noise level.

*The model simulation results showed that no model including an asymmetry between positive and negative outcomes, scaled by parameter a, was recoverable. This result is surprising, given that (i) this type of specification is frequently used in the field of reinforcement learning, where a positivity bias (a > 0) has consistently been reported (Palminteri & Lebreton, 2022) and (ii) analytically, the asymmetry parameter a can be distinguished from the other model parameters when the outcome sequence is designed for this purpose.*

*Using our model simulation results, we studied the influence of model specification and parameter values on the identifiability of models that included a. To do so, we ranked our simulated subjects along the factor dimension of interest and compared recovery across the different models including a. Specifically, for each of these models, we defined identifiability as selection of the model by random-effects Bayesian model comparison, relative to its direct counterpart without the asymmetry effect. We used this restricted model space for model comparison because the closest model that didn’t include an asymmetry term had been systematically selected in our initial analysis.*

*We found that the primary factors determining model identifiability were (i) the strength of the asymmetry bias, defined as the distance of parameter a from neutral value 1, |a-1| and (ii) the absolute asymmetry deviation, defined as the strength of the bias multiplied by |ω_f_|. Comparing models M7, M8, M9 and M10 two by two, we also found that other model specifications influenced model recovery:*

- *Adding an EV accumulation term increased the minimum asymmetry-bias, or absolute-asymmetry-deviation, threshold required for model recovery.*
- *Adding a reciprocal effect of mood, scaled by parameter δ, further increased this threshold and generated intervals of non-identifiability above the threshold, producing non-monotonic patterns that suggest more complex interactions.*

*We also noted that, in noisy simulations, models with asymmetry became partially identifiable (main manuscript, figure 3). Moreover, in our simulations with continuous outcomes, the asymmetry parameter recovery correlations seemed to increase as compared to binary outcomes (S1 Fig.; main manuscript, Fig. 2).*

*In conclusion, models that included an asymmetry between negative and positive outcomes were not intrinsically unrecoverable. Rather, the lack of identifiability that we observed appears to result from the interaction of several factors, including generative parameter values, model specifications, binary outcomes and noise level.*

Regarding the more specific remark about mixed lotteries, we agree that using this type of design could help identify an indifference point that could subsequently be used in mood models, under the assumption that loss aversion has a similar magnitude in choice behaviour and mood computation. We added this remark in the main text discussion (lines 450-456):

*Dedicated analyses showed that the identifiability of this parameter depends primarily on the absolute magnitude of the asymmetry, but also interacts with other model specifications, such as the inclusion of expected value accumulation and reciprocal effect of mood (S3 Appendix). One way to circumvent this identifiability issue could be to use mixed lotteries in which gains and losses are weighted in the same trials, allowing an indifference point to be fitted directly to choices and an asymmetry parameter to be inferred from it, that could in turn inform a mood model.*

*4. Table 2 reports a negative mean value for the \delta parameter (mood’s effect on perceived feedback) in the Riven task. This seems to contradict the assumption that “good mood induces a rosy outlook.” If correct, this counter-intuitive result merits further explanation: why does positive (negative) mood appear to decrease (increase) the subjective perception of feedback in the Riven task?*

Even though group mean signs differed numerically, we did not find any significant between-groups difference regarding the reciprocal weight of mood, δ. We reported this in the main text results section (lines 394-6):

*The reciprocal-influence parameter δ, which quantifies the effect of mood on feedback perception, did not differ between task (F(2,83) = 2.31, p = .11) and its global group mean did not differ from zero (t(235) = -1.11, p = .27).*

This being said, we agree that the “rosy outlook” effect was the cognitive motivation for this model specification, as proposed by Eldar and colleagues. When choosing not to restrict δ to positive values in our model fitting procedure, we departed from this framework. It turned out that individually fitted values for δ were widely distributed, while the group mean did not differ significantly from zero.

A simple cognitive interpretation for negative values of δ is a contrast effect: a given outcome seems less rewarding relatively to one’s good mood (and the rewards previously accumulated). We added this point to the main text discussion (lines 523-531):

*Notably, the weight of mood on subjective feedback δ did not differ across tasks, but was widely distributed across participants, with a group mean not significantly different from zero. Positive values of δ are indicative of a mood-congruent bias, resulting in a “rosy outlook” when mood is high and a “gloomy outlook” when it is down, and might promote mood swings by amplifying small fluctuations. Negative values of δ, by contrast, can be interpreted as a contrast effect, with higher mood leading to an attenuation of the positive valuation of better outcomes, and might reflect a tendency towards regulation around an equilibrium.*

*5. The paper should explicitly specify which part of the dataset were previously used in Heerema et al. (2023). This transparency is essential for readers to assess the novelty and independence of the current findings.*

We clarified this point regarding the reference to the economic choice dataset analysed in Heerema et al., 2023 (lines 307-310):

*The main purpose of these economic choices was to assess the effect of mood on decision making (see ^25^ for the results, where the analysed dataset includes only the quiz task). Therefore, they are not analysed further in this paper.*

*6. The abstract states that "computational models of mood dynamics show robust validity and satisfactory test-retest reliability", yet the the discussion section specifically highlights support for the MAGNETO model without referencing other published models of mood (lines 555-556). To provide readers with a clearer understanding of how the current findings relate to existing models, the authors should explicitly map the eight representative models of interest (lines 155–156) to their corresponding published counterparts.*

We want to thank the reviewer for this suggestion, that helped clarify the modelling section. We added a dedicated column in the model table and a paragraph describing the evolution of the field (lines 198-204):

*This model space is conceived as a summary of previous research focusing on explicit mood modelling in laboratory-based studies. In a landmark study, Rutledge and colleagues introduced the leaky accumulation model of affect integrating outcomes, expected values and reward prediction errors^19^. Eldar and colleagues added a reciprocal effect of mood on outcome perception, first in a multiplicative^23^, then in an additive form^14^. We introduced an asymmetric effect of negative and positive outcomes in a previous study^24^, as well as a time drift as a nuisance term. Notably, a negative drift in mood during such tasks has since been shown to be a robust contextual effect.^37^*

| Model Number | Model Name | Asymmetry between negative and positive outcomes ($a$) | Reciprocal effect of mood on feedback ($\delta$) | Impact of expected value on mood ($\omega_{\boldsymbol{e}}\boldsymbol{)}$ | Original publication |
| --- | --- | --- | --- | --- | --- |
| M1 | Control1 | Control model where mood is a linear function of time | | |  |
| M2 | Control2 | Control model where only the last feedback is considered | | |  |
| M3 | Asy-Rec-Exp- | 0 | 0 | 0 |  |
| M4 | Asy-Rec-Exp+ | 0 | 0 | 1 | Rutledge et al. 2014 |
| M5 | Asy-Rec+Exp- | 0 | 1 | 0 |  |
| M6 | Asy-Rec+Exp+ | 0 | 1 | 1 | Eldar & Niv 2015, Eldar et al. 2016 |
| M7 | Asy+Rec-Exp- | 1 | 0 | 0 |  |
| M8 | Asy+Rec-Exp+ | 1 | 0 | 1 |  |
| M9 | Asy+Rec+Exp- | 1 | 1 | 0 | Vinckier et al. 2018 |
| M10 | Asy+Rec+Exp+ | 1 | 1 | 1 |  |

**Table 1.** **Model space.** Two control models and 8 models of interest were studied in terms of model identifiability and parameter recovery.

We also suppressed the sentence that referenced to the MAGNETO model in the discussion, as we agree this introduced a distraction from the main objectives of this study.

*Reviewer #3: This manuscript addresses the important question of whether computational models of mood dynamics show robust validity and test-retest reliability. These models are increasingly used in the field of computational psychiatry, but this is the first detailed test of model and parameter recovery across multiple tasks using multiple mood models. The authors report promising results across multiple tasks and samples. They also identify multiple important issues that will be very valuable for researchers using these tools, including issues with asymmetric feedback weights and linear interpolation of mood ratings. Their overall results suggest that these tasks and models have the kind of characteristics that would make them valuable for computational phenotyping and clinical use. There are a couple of issues that, once resolved, would make their contribution more valuable.*

We want to thank the reviewer for their encouraging assessment and helpful feedback.

*-The authors suggest that reliability is substantially lower when using infrequent ratings. However, it is unclear whether this is simply because a much smaller number of ratings are being fit. If the number of ratings is matched across simulations with different rating frequencies, how is reliability affected?*

We thank the reviewer for this question, which probed us to clarify this part of the simulation results. Indeed, our analyses showed that the primary factor that impaired model recovery was the total number of ratings, rather than the rating frequency. From an experimenter’s point of view, doubling task duration necessarily seems costlier than doubling mood rating frequency. Nevertheless, this is an important point to ponder when designing this type of experiment, and this result can influence task design decisions. We therefore added it to the main text (lines 253-259 and Fig. 4):

*When mood ratings were downsampled, simulating a design in which mood was not probed on every trial, model identifiability decreased significantly. Specifically, the identifiability of the model variants that included an effect of mood on feedback perception (δ) was impaired, as their simpler variant (without this reciprocal effect) was selected by model comparison. However, keeping the number of mood ratings constant while varying their frequency tended to improve model recovery, suggesting that the observed decrease in model identifiability was attributable to the smaller number of samples rather than to lower rating frequency per se (Fig. 4). We also noted that, for a fixed number of mood ratings, model recovery was strongly determined by the temporal coverage of the rating schedule. Uniformly spaced ratings outperformed unconstrained random sampling, likely because random schedules produced clusters and long gaps that reduced observability of the latent mood trajectory, and are therefore presented as the main results (but see S3 Fig. and S4 Fig. for results with random sampling).*

**Figure 4. Effect of the number and frequency of mood ratings on model identifiability**. (A) Mean recovery exceedance probability as a function of the proportion of trials in which mood ratings are collected, in two situations: fixed total trial number (red) and fixed total mood rating number (grey pink). Dotted lines denote linear interpolation for missing data. (B) Confusion matrix (exceedance probability of each model given the model that generated the data) when mood ratings are collected every 4 trials out of a total of 75 trials, without (top) and with (bottom) linear interpolation for missing data. (C) Confusion matrix when mood ratings are collected every 4 trials for a total of 75 mood ratings (300 trials), without (top) and with (bottom) linear interpolation for missing data.

*-The authors clearly show that linear interpolation of missing ratings is a bad idea for these three tasks. However, it is unclear exactly why and whether there are situations in which linear interpolation makes sense. If the winning models are used to make prediction for intermediate timepoints between ratings in the actual data, these can be compared to interpolated ratings for the actual data. It is possible that there is little correlation between the actual ratings observed and those interpolated when discarding data in the ways suggested. There should be a correlation between actual ratings and the model predictions for those timepoints if the ratings are discarded and the models fit on the remaining ratings. It would be helpful for readers to understand whether there is some situation in which simulations show that linear interpolation is helpful, potentially with a variant of one of these tasks.*

We are not sure to understand exactly the reviewer’s suggestion. Following their reasoning, we performed the following analysis: following the same procedure with our experimental dataset as we did with our simulations, we downsampled the mood rating timeseries, used linear interpolation to estimate missing data points, fitted our selected model M5 to the downsampled mood ratings, and used this fitted model to compute model predictions for missing data points. We then computed the correlations between true discarded ratings and linear interpolation estimates, as well as between true discarded ratings and M5 model predictions. We did so for downsampling rates of 1/2, 1/3 and 1/4 (corresponding to mood the same frequency of mood rating in a trial-by-trial design), as we did for simulations presented in the main text. We then compared correlation estimates for every situation (figure below). We found that for the lottery task, the correlations between true ratings and interpolation estimates were higher than the correlations between true ratings and model predictions, and that this patter was reversed for the riven task. On the other hand, there were no difference between correlation coefficients for the quiz task and when task-groups were pooled together.

We are unsure about the potential interpretations of this result. It seems to show that, when information about some trials is missing, model predictions don’t perform better than interpolation, even when they are informed by the result of the model selection performed with complete data.

Regarding the specific point about potential situations in which interpolation might be helpful: from our results, the only situation in which this might be the case was when mood ratings were not only sparse, but also randomly distributed in an unconstrained manner. In this case (S3 Fig, reproduced below), interpolation produced a better mean recovery exceedance probability (panel A); however, this result has to be tempered by the bias to complex models introduced by interpolation (panel B and C), which might further impair interpretability.

**S3 Figure. Effect of the number and frequency of mood ratings on model identifiability for randomly placed ratings.** (A) Mean recovery exceedance probability as a function of the proportion of trials in which mood ratings are collected, in two situations: fixed total trial number (red) and fixed total mood rating number (grey pink). Dotted lines denote linear interpolation for missing data. (B) Confusion matrix (exceedance probability of each model given the model that generated the data) for 19 randomly placed mood ratings out of a total of 75 trials, without (top) and with (bottom) linear interpolation for missing data. (C) Confusion matrix for a total of 75 randomly placed mood ratings (300 trials), without (top) and with (bottom) linear interpolation for missing data.

*-The lack of replication of the prediction error result with the lottery task is interesting. The extra complexity of this version makes sense as a possible reason. Any timing differences between the designs should also be noted including the duration of any delays and time between trials. Average risk taking in the gain, mixed, and loss trials should also be reported because there could be significant behavioral differences to what has been observed in previous studies. It should also be noted in the discussion that ratings are collected after every trial in this study, and this is a key difference compared to much of the literature where ratings are not collected on every trial. Asking about mood on every trial may encourage participants to rely more on memory of the most recent event, and not the overall cumulative impact of expectations for multiple trials.*

We added a dedicated SI file with details about the lottery task design used in this study (S2 Appendix). In particular, we described all the differences we could find with the published design (per Rutledge et al., 2014):

*The task was adapted from Rutledge et al. (2014), with several differences. First, participants had 10 s to make their choice, whereas Rutledge et al. used a 3-s choice time limit. Second, the feedback timing differed: in the present task, the selected option remained visible for 1 s and the outcome was then displayed for 1 s, whereas in Rutledge et al., certain choices remained on the screen for 7 s and lottery choices remained on the screen for 6 s before the outcome was revealed for 1 s. Third, the present task used a fixed 1-s fixation period before each trial; Rutledge et al. used a 3–11 s jittered intertrial interval with a saturated Gamma distribution in their fMRI experiment design, no specific description was specified for behaviour-only experiments. Fourth, mood ratings were collected after every trial, whereas Rutledge et al. collected happiness ratings after every two to three trials. Finally, the wording of the affective rating differed: participants in the present study rated their mood on a scale from “bad mood” to “good mood,” whereas Rutledge et al. asked participants how happy they were at that moment on a scale from “very unhappy” to “very happy.”*

Note that in this description, we compared our task design to the detailed task description found in the Supplementary Methods sections of (Rutledge et al., 2014), which refers to their MRI experiment. Therefore, there might be some other differences with other versions of the task used by the same group, especially regarding jittered intertrial intervals. However, we note that this team has replicated the RPE result across many different settings (MRI, lab-based behavioural experiments, smartphone app), providing evidence that minor changes in task timing should not influence the result.

We also reported choice behaviour for this task in the main text (lines 328-330):

*In the lottery task, bet acceptance rates depended on the gamble type (one-sample t-tests, all p < .001): mean ±SD in the loss, mixed and gain trials were respectively .37±.24, .53±.23, and .70±.24.*

Finally, we acknowledged the potential effect of mood rating frequency on results in the main text discussion (lines 511-517):

*Aside from a potential issue of statistical power, the most plausible explanation relates to a significant alteration in our experimental design, notably the introduction of an orthogonal task (economic choice) between feedback and mood assessment. This modification was intentionally implemented to weaken the direct association participants might perceive between feedback and subsequent mood evaluation. Another significant difference from the published design is the inclusion of mood ratings on every trial, which may have narrowed the participants’ focus to the immediately preceding trial and interacted with the way outcomes and prediction errors were accumulated.*

*-Simulations do not seem to have any noise in them. It would be useful to have simulations with realistic amounts of noise (estimated from the actual data) to see how this impacts key findings, including parameter reliability for the distribution of parameter estimates observed from the data.*

We agree that the absence of noise in our original manuscript considerably restricted the scope of the findings. In the revised manuscript, we included a new section dedicated to the analysis of the effect of noise on model and parameter recovery, with a focus on realistic noise level estimated from the empirical dataset (lines 222-250 and Fig. 3):

*In order to assess the effect of noisy mood ratings, we incorporated a reliability parameter α controlling the relative contribution of the model-predicted signal and random response variability. Specifically, after mapping the latent model prediction onto the bounded response scale, the observed response was generated as a mixture of this bounded signal and a random contaminating component:*

$$y_{t}=\alpha m_{r(t)}+\left( 1-\alpha\right)\epsilon_{t}$$

*where ϵ_t_ was sampled from a uniform distribution over the same interval, ϵ_t_ ∼ U (−1,1), and α∈ [0,1]. This formulation ensures that simulated observations remain within the admissible response range for all values of α. The parameter α can be interpreted as a reliability or signal-preservation parameter: when α = 1, responses are fully determined by the model-predicted signal, whereas when α = 0, responses are independent of the model and uniformly distributed over the response scale. Intermediate values correspond to partial corruption of the model-predicted signal by bounded random variability.*

*Systematically assessing model and parameter recovery with varying α values, we found that global model identifiability plateaus until α values around 0.5 (corresponding to a signal-to-noise ratio of 1), before decreasing sharply. By contrast, parameter recovery follows a seemingly linear decrease until α values around 0.3 before collapsing (Fig 3A and 3D).*

*To assess the global reliability of mood ratings in the experimental dataset, we estimated the empirically predictable variance using a global mixed-effects linear model that included lagged Volterra predictors and autoregressive terms for past mood ratings. This analysis yielded a R2 of 0.865, corresponding to an estimated reliability parameter α = 0.717, with the remaining unexplained variance taken as a plausible estimate of empirical noise.*

*Examining confusion matrices around this value, we found that the model recovery pattern remained close to the noiseless reference, with perfect identification of models that didn’t include an asymmetry parameter (all xp > .99, Fig. 3A-C). Consistent with the global pattern, parameter recovery was more impacted by noise than model identifiability at the empirical estimate. Recovery remained high ω for parameters (r > 0.67, 95%CI lower bound > .59), but fell to intermediate levels for the decay factor γ (0.51 < r < 0.94), which became sensitive to the presence of the parameter that governs the reciprocal effort of mood on feedback perception, δ (Fig. 3D-F). The two remaining parameters a and δ showed low recoverability (r < .40, 95%CI higher bound < .50).*

**Figure 3. Effect of mood rating reliability on model and parameter recovery.** (A) Mean model recovery exceedance probability as a function of decreasing signal weight. (B) Confusion matrix (exceedance probability of each model given the model that generated the data) of the full model space for the experimental noise estimate (α = 0.72). (C) Confusion matrix of the restricted model space for the experimental noise estimate. (D) Mean parameter recovery correlation as a function of decreasing signal weight. (E) Same-parameter recovery across models for the experimental noise estimate (α = 0.72). (F) Selected model cross-parameter correlation for the experimental noise estimate.

*-The importance of controlling the precise feedback sequence does not come across clearly. Particularly since it doesn’t seem to make a difference for the results, it would be useful to make clear whether any simulations suggest that it would be valuable given what has been learned here.*

We thank the reviewer for raising this point, as we agree it was not properly detailed in the original manuscript. We detailed the motivation to test the sensitivity of results to feedback sequence specifics in the introduction (lines 109-118):

*Some models added a reciprocal effect of mood on outcome perception, such that good mood induces a “rosy outlook”. Given that individuals may integrate identical feedback sequences differently (leading to distinct mood trajectories), these models include free parameters that characterize the temporal dynamics of mood responses to past outcomes. Critically, reciprocal interactions make the model nonlinear: the current mood state influences how subsequent feedback is integrated, so small differences in early event sequences can produce markedly different long-term trajectories. This raises an important methodological concern: to what extent do the free parameters derived from participants' mood ratings genuinely reflect stable individual traits, rather than being driven by incidental differences in the feedback sequence they experienced?*

*Minor comments*

*-The opening sentence of the abstract refers to mood as being about outcomes related to intended outcomes. It is unclear what ‘intended outcomes’ means because presumably mood could change due to events that don’t have intended outcomes, like when someone reads the news.*

We acknowledge that this formulation was confusing. We simplified this sentence, given that its point was simply to broadly introduce the type of conception of mood that underly the computational models used in this study, rather than to advocate for a particular model regarding which type of actions or outcomes primarily drive mood fluctuations (lines 18-19):

*Mood can be understood as an affective state resulting from the integration, over time, of positive and negative outcomes.*

*-Mood ratings are said to be bounded by -1 and 1, but figure 4 shows z-scored ratings and then mean mood that is bounded by 0 and 1. It should be clear in all places whether analyses are about z-scored mood and when mood is not transformed and what the bounds are.*

We clarified mood ratings scaling in the captions of figures 6, 7 and 8 and table 2. We used relatively standard conventions:

- by default, mood ratings are scaled between 0 and 1 (as for mean mood in Fig. 6 [Fig. 4 in the original manuscript])
- in all simulations and model-based analyses, mood ratings are scaled between -1 and 1 in order to avoid asymmetry between positive and negative outcome integration
- we specified whenever z-scores are used, i.e., mainly in linear and model-free analyses where variance normalising across subjects is useful for group-level summaries.

*-Is wt for time the same as the drift in the paper by Jangraw that uses lottery tasks? The connection between what is found here and that paper should be discussed.*

We added a mention to (Jangraw et al., 2023) in the paragraph where we describe the construction of the model space with respect to previous literature (lines 198-204):

*This model space is conceived as a summary of previous research focusing on explicit mood modelling in laboratory-based studies. In a landmark study, Rutledge and colleagues introduced the leaky accumulation model of affect integrating outcomes, expected values and reward prediction errors^19^. Eldar and colleagues added a reciprocal effect of mood on outcome perception, first in a multiplicative^23^, then in an additive form^14^. We introduced an asymmetric effect of negative and positive outcomes in a previous study^24^, as well as a time drift as a nuisance term. Notably, a negative drift in mood during such tasks has since been shown to be a robust contextual effect.^37^*

And in the discussion (lines 539-541):

*More generally, this result is consistent with previous findings that a negative time drift is a consistent and robust driver of mood during cognitive tasks.^37^*

*-In the table 2 caption and potentially elsewhere, it would be helpful to name each of the parameters so that readers are not confused about which one is for drift, for example. These should also be compared to published studies to the extent possible. Are these w0 the same as previously observed? These values are consistent with a -1 to 1 scale but a previous figure shows mean values that seem to be on a 0 to 1 scale. They should also be compared across the different tasks. For example, the forgetting factor seems to differ.*

We added a cognitive interpretation for every parameter in table 2:

| Parameter | $\omega_{\boldsymbol{0}}$ | $\omega_{\boldsymbol{f}}$ | $\omega_{\boldsymbol{t}}$ | $\gamma$ | $\delta$ |
| --- | --- | --- | --- | --- | --- |
| Interpretation | Mood baseline | Sensitivity to outcomes | Time drift | Forgetting factor | Reciprocal effect of mood on outcome perception |
| Lottery | 0.25 (0.39) | 0.060 (0.15) | -0.046 (0.20) | 0.78 (0.19) | 0.10 (0.93) |
| Quiz | 0.20 (0.29) | 0.032 (0.047) | -0.036 (0.12) | 0.78 (0.20) | 0.016 (1.34) |
| Riven | 0.20 (0.34) | 0.063 (0.089) | -0.056 (0.14) | 0.69 (0.23) | -0.21 (0.85) |

**Table 2. Summary statistics of fitted parameter distributions in each task (first session).** Reported figures are mean (standard deviation), for each parameter and each task. All values are relative to mood ratings scaled between -1 and 1 and a fixed sigmoid observation mapping.

We added references to published models in table 1:

| Model Number | Model Name | Asymmetry between negative and positive outcomes ($a$) | Reciprocal effect of mood on feedback ($\delta$) | Impact of expected value on mood ($\omega_{\boldsymbol{e}}\boldsymbol{)}$ | Original publication |
| --- | --- | --- | --- | --- | --- |
| M1 | Control1 | Control model where mood is a linear function of time | | |  |
| M2 | Control2 | Control model where only the last feedback is considered | | |  |
| M3 | Asy-Rec-Exp- | 0 | 0 | 0 |  |
| M4 | Asy-Rec-Exp+ | 0 | 0 | 1 | Rutledge et al. 2014 |
| M5 | Asy-Rec+Exp- | 0 | 1 | 0 |  |
| M6 | Asy-Rec+Exp+ | 0 | 1 | 1 | Eldar & Niv 2015, Eldar et al. 2016 |
| M7 | Asy+Rec-Exp- | 1 | 0 | 0 |  |
| M8 | Asy+Rec-Exp+ | 1 | 0 | 1 |  |
| M9 | Asy+Rec+Exp- | 1 | 1 | 0 | Vinckier et al. 2018 |
| M10 | Asy+Rec+Exp+ | 1 | 1 | 1 |  |

**Table 1.** **Model space.** Two control models and 8 models of interest were studied in terms of model identifiability and parameter recovery.

We added a between-task comparison of computational parameters for the selected model in the results section of the main text (lines 392-398):

*The forgetting factor γ differed between tasks (F(2,99) = 5.98, p = .004); specifically, post-hoc comparisons indicated γ was lower in the Riven task than in both the quiz task (p = .004) and lottery task (p = .02). The reciprocal-influence parameter δ, which quantifies the effect of mood on feedback perception, did not differ between task (F(2,83) = 2.31, p = .11) and its global group mean did not differ from zero (t(235) = -1.11, p = .27). The weight on feedback accumulation, ω_f_, also showed a main effect of task (F(2,64) = 7.32, p = .001) with lower values in the quiz task than in the Riven task (post-hoc comparison, p = .003).*

Finally, we added a discussion of computational parameter differences and interpretations across tasks and studies (main text discussion, lines 517-541):

*From a computational psychiatry perspective, subject-level model parameter estimates, often referred to as “computational phenotype”, are key outcomes. A central hope in the field is that these estimates will enable researchers to shed a new light on inter-individual differences, diagnosis and clinical prediction^34,42^. In this regard, it is necessary to quantitatively assess the sources of variation in parameter estimates. One potential source is the particular task used for model fitting and parameter estimation. Our results across three different tasks were mixed. We found that in the Riven task, the forgetting factor γ was lower than in the lottery and quiz tasks, indicating that fewer previous trials contributed to mood at a given trial. Notably, the weight of mood on subjective feedback δ did not differ across tasks, but was widely distributed across participants, with a group mean not significantly different from zero. Positive values of δ are indicative of a mood-congruent bias, resulting in a “rosy outlook” when mood is high and a “gloomy outlook” when it is down, and might promote mood swings by amplifying small fluctuations. Negative values of δ, by contrast, can be interpreted as a contrast effect, with higher mood leading to an attenuation of the positive valuation of better outcomes, and might reflect a tendency towards regulation around an equilibrium.*

*Comparing computational parameter estimates with previous literature is challenging, given that values are always relative to other model specifications, which is why few studies actually report them. This is especially true for feedback sensitivity, which is often estimated in models that also include accumulation terms for EV and RPE, making comparison with our selected model irrelevant. However, we note that baseline mood values reported in ^43^ are similar to our findings, with a mean around 60% of scale range. Forgetting factor values are reported in many studies that mainly use variants of the lottery task; these reported values are in the same range as our estimates.^19,21,44–48^ One study also reports a time drift parameter between -2% and -4% of scale range, similar to our finding.^48^* *More generally, this result is consistent with previous findings that a negative time drift is a consistent and robust driver of mood during cognitive tasks.^37^*

*-Are the parameters correlated across tasks in participants that have completed two tasks? The means suggest differences, but the correlations across task should be included. Especially since w0 is not correlated in time in gambling but is for the other tasks, a careful comparison of w0 across tasks collected in the same session would be useful to understand why this parameter seems to be more stable in some tasks.*

We reported this result as S5 Fig:

**S5 Figure. Cross-task parameter stability for tasks completed in a same experimental session.** *Correlation between the parameters of the selected mood model, that was fit separately to two tasks that were completed in a same experimental session. X-axis denotes parameter estimates in the Riven task. Y-axis denotes parameter estimates in the quiz task (first row) or in the lottery task (second row). No subject completed both the lottery and quiz tasks. Consistently with the main results, the most stable parameters were baseline mood ω_0_ and feedback accumulation sensitivity ω_f_. The correlation is only significant for ω_f_ values between the Riven and quiz tasks, which may be related to similarities in their designs. The lack of correlation between baseline mood ω_0_ estimates might relate to the negative time drift frequently observed during this type of tasks, which could dissociate baseline estimates when two tasks are completed sequentially within the same experimental session.*

These results are consistent with our main results (main manuscript, Fig. 7), which showed that the most stable parameters were baseline mood ω_0_ and feedback accumulation sensitivity ω_f_. Nevertheless, the correlation is only significant for ω_f_ values between the Riven and quiz tasks, which may be related to similarities in their designs: both tasks used binary outcomes, temporally structured feedback sequences, and a sense of agency rather than chance, in contrast with the lottery task.
