# Supplementary material for: Assessing the validity and reliability of computational phenotyping of mood
Source: PLoS Comput Biol. 2026 Aug 3;22(8):e1014597. doi: 10.1371/journal.pcbi.1014597 (PMC13456276; doi:10.1371/journal.pcbi.1014597)
Supplement: Peer review history

# Peer review: Decision Letter 0

**DOI:** 10.1371/journal.pcbi.1014597.r001

**Authors:** Natalia Komarova, Christoph Mathys

2 Feb 2026

PCOMPBIOL-D-25-02233

Assessing the validity and reliability of computational phenotyping of mood

PLOS Computational Biology

Dear Dr. Benhamou,

Thank you for submitting your manuscript to PLOS Computational Biology. After careful consideration, we feel that it has merit but does not fully meet PLOS Computational Biology's publication criteria as it currently stands. Therefore, we invite you to submit a revised version of the manuscript that addresses the points raised during the review process.

Please submit your revised manuscript by Apr 04 2026 11:59PM. If you will need more time than this to complete your revisions, please reply to this message or contact the journal office at ploscompbiol@plos.org. When you're ready to submit your revision, log on to https://www.editorialmanager.com/pcompbiol/ and select the 'Submissions Needing Revision' folder to locate your manuscript file.

Please include the following items when submitting your revised manuscript:

* A letter that responds to each point raised by the editor and reviewer(s). You should upload this letter as a separate file labeled 'Response to Reviewers'. This file does not need to include responses to formatting updates and technical items listed in the 'Journal Requirements' section below.

* A marked-up copy of your manuscript that highlights changes made to the original version. You should upload this as a separate file labeled 'Revised Manuscript with Track Changes'.

* An unmarked version of your revised paper without tracked changes. You should upload this as a separate file labeled 'Manuscript'.

If you would like to make changes to your financial disclosure, competing interests statement, or data availability statement, please make these updates within the submission form at the time of resubmission. Guidelines for resubmitting your figure files are available below the reviewer comments at the end of this letter

We look forward to receiving your revised manuscript.

Kind regards,

Christoph Mathys

Academic Editor

PLOS Computational Biology

Natalia Komarova

Section Editor

PLOS Computational Biology

**Journal Requirements:**

1) Please ensure that the CRediT author contributions listed for every co-author are completed accurately and in full.

At this stage, the following Authors/Authors require contributions: Pablo Carrillo, Roeland Heerema, Marc Benhamou, Jean Daunizeau, Mathias Pessiglione, and Fabien Vinckier. Please ensure that the full contributions of each author are acknowledged in the "Add/Edit/Remove Authors" section of our submission form.

The list of CRediT author contributions may be found here: https://journals.plos.org/ploscompbiol/s/authorship#loc-author-contributions

2) Please provide an Author Summary. This should appear in your manuscript between the Abstract (if applicable) and the Introduction, and should be 150-200 words long. The aim should be to make your findings accessible to a wide audience that includes both scientists and non-scientists. Sample summaries can be found on our website under Submission Guidelines:

https://journals.plos.org/ploscompbiol/s/submission-guidelines#loc-parts-of-a-submission

3) Please upload all main figures as separate Figure files in .tif or .eps format. For more information about how to convert and format your figure files please see our guidelines:

https://journals.plos.org/ploscompbiol/s/figures

4) We have noticed that you have uploaded Supporting Information files, but you have not included a list of legends. Please add a full list of legends for your Supporting Information files after the references list.

5) Some material included in your submission may be copyrighted. According to PLOSu2019s copyright policy, authors who use figures or other material (e.g., graphics, clipart, maps) from another author or copyright holder must demonstrate or obtain permission to publish this material under the Creative Commons Attribution 4.0 International (CC BY 4.0) License used by PLOS journals. Please closely review the details of PLOSu2019s copyright requirements here: PLOS Licenses and Copyright. If you need to request permissions from a copyright holder, you may use PLOS's Copyright Content Permission form.

Please respond directly to this email and provide any known details concerning your material's license terms and permissions required for reuse, even if you have not yet obtained copyright permissions or are unsure of your material's copyright compatibility. Once you have responded and addressed all other outstanding technical requirements, you may resubmit your manuscript within Editorial Manager.

Potential Copyright Issues:

- Figure 1. Please confirm whether you drew the images / clip-art within the figure panels by hand. If you did not draw the images, please provide (a) a link to the source of the images or icons and their license / terms of use; or (b) written permission from the copyright holder to publish the images or icons under our CC BY 4.0 license. Alternatively, you may replace the images with open source alternatives. See these open source resources you may use to replace images / clip-art:

- https://commons.wikimedia.org

- https://openclipart.org/.

**Reviewers' comments:**

Reviewer's Responses to Questions

**Comments to the Authors:**

**Please note here if the review is uploaded as an attachment.**

Reviewer #1: The manuscript presents a useful characterization of common models of mood in terms of identifiability, and an empirical test of the stability of their parameters in the context of three mood manipulation tasks. The effects of outcomes on mood are found to be stable and replicable across tasks. Other aspects such as the effect of mood on subjective outcome, and the asymmetry in mood updates, were less identifiable. The work makes a meaningful contribution to the literature on computational models of mood, but the following issues need to be addressed:

It needs to be acknowledged that the choice of tasks may have a lot to do with the observed results. The tasks represent a particular approach to manipulating mood, which uses relatively small recurring outcomes and does not engage learning (learning likely happens in some ways but it is not quantified or controlled). This approach is popular, and this makes the authors' choice reasonable, but the results have to be interpreted in this light because (1) effects of mood on subjective outcome have not only but primarily been demonstrated when mood was manipulated using big singular outcomes, and (2) by some accounts mood is driven by learning from reward prediction errors, rather than by the reward prediction errors themselves. A different task might therefore have led to more stable and identifiable effects of mood on subjective outcome, and to a more identifiable role for EV in shaping mood.

The unidentifiability of an asymmetry in mood updates is currently not convincingly explained. In theory, when all outcomes equal zero, then only w_0 can have an effect on predicted mood, so this parameter should be dissociable from both w_f and alpha. Furthermore, when all outcomes are negative, then only w_0 and w_f can influence mood and not alpha. Together, these two dissociations should enable identifying the three parameters. Likewise, the results of the parameter recovery test do not seem to support the lack of dissociation proposed by the authors, as the spurious correlations between alpha and w_0/w_f are fairly low.

Presumably the surprising results regarding interpolation reflect the unsuitability of the linear functional form of the interpolation. It could be worth while to test other forms such as Gaussian filtering or spline interpolation.

Line 169: was some specific noise distribution assumed around the predicted m?

Reviewer #2: This study investigates computational models of mood dynamics during decision-making, and introduces the novel Riven task designed to control feedback sequences while preserving participants' sense of agency over outcomes. The work addresses three key objectives: (1) examining how feedback sequences influence the free parameters governing short-term mood fluctuations, (2) assessing the robustness of existing mood models in terms of model identification and parameter recovery, and (3) evaluating test-retest consistency by repeating the task after a two-week interval. The authors demonstrate that a representative set of mood dynamics models - previously employed in the literature - exhibit robust validity and satisfactory test-retest reliability, though some models proved non-identifiable. Building on the authors' previous work (Vinckier et al., 2018; Heerema et al., 2023; Pessiglione et al., 2023), this study makes a valuable contribution to the literature on mood dynamics. While the manuscript is well-structured, addressing the following points could further enhance its clarity and rigour:

1. The economic choice task, though not the primary focus, may exert unintended influence on mood verbal reports. Even in the absence of explicit feedback, performing the task could evoke implicit expectations about future gains or losses. These expectations, in turn, may independently modulate participants’ mood states, adding an additional layer of complexity to the estimation of the expected value (EV), beyond the challenges already raised in the discussion section (lines 537–552). To what extent might this unintended influence bias key findings, such as the selection of model 5 over models including the EV term?

2. The finding that EV does not influence mood in the tested models is surprising, particularly in light of the well-documented link between Reward Prediction Error (RPE) and affective valence. The authors acknowledge that EV estimation may be challenging in the quiz and Riven tasks, but less so in the gambling task. The authors could supplement the model-free analyses with an examination of RPE (= feedback – EV) effects on mood. This could be visualised in Figure 4, providing a more comprehensive understanding of the relationship between RPE and mood fluctuations, at least within the gambling task.

3. The asymmetry term appears to be closely related to loss aversion, a widely recognised cognitive bias in economic and psychological models. Could the identifiability of the models including an asymmetry term be improved by employing mixed lotteries (combining gains and losses) instead of the pure gain/loss lotteries used in this study?

4. Table 2 reports a negative mean value for the \delta parameter (mood’s effect on perceived feedback) in the Riven task. This seems to contradict the assumption that “good mood induces a rosy outlook.” If correct, this counter-intuitive result merits further explanation: why does positive (negative) mood appear to decrease (increase) the subjective perception of feedback in the Riven task?

5. The paper should explicitly specify which part of the dataset were previously used in Heerema et al. (2023). This transparency is essential for readers to assess the novelty and independence of the current findings.

6. The abstract states that "computational models of mood dynamics show robust validity and satisfactory test-retest reliability", yet the the discussion section specifically highlights support for the MAGNETO model without referencing other published models of mood (lines 555-556). To provide readers with a clearer understanding of how the current findings relate to existing models, the authors should explicitly map the eight representative models of interest (lines 155–156) to their corresponding published counterparts.

Reviewer #3: This manuscript addresses the important question of whether computational models of mood dynamics show robust validity and test-retest reliability. These models are increasingly used in the field of computational psychiatry, but this is the first detailed test of model and parameter recovery across multiple tasks using multiple mood models. The authors report promising results across multiple tasks and samples. They also identify multiple important issues that will be very valuable for researchers using these tools, including issues with asymmetric feedback weights and linear interpolation of mood ratings. Their overall results suggest that these tasks and models have the kind of characteristics that would make them valuable for computational phenotyping and clinical use. There are a couple of issues that, once resolved, would make their contribution more valuable.

-The authors suggest that reliability is substantially lower when using infrequent ratings. However, it is unclear whether this is simply because a much smaller number of ratings are being fit. If the number of ratings is matched across simulations with different rating frequencies, how is reliability affected? Analyses should be shown both from the kinds of simulations already used and also using models fit to the actual data so that the distributions of parameters match those observed.

-The authors clearly show that linear interpolation of missing ratings is a bad idea for these three tasks. However, it is unclear exactly why and whether there are situations in which linear interpolation makes sense. If the winning models are used to make prediction for intermediate timepoints between ratings in the actual data, these can be compared to interpolated ratings for the actual data. It is possible that there is little correlation between the actual ratings observed and those interpolated when discarding data in the ways suggested. There should be a correlation between actual ratings and the model predictions for those timepoints if the ratings are discarded and the models fit on the remaining ratings. It would be helpful for readers to understand whether there is some situation in which simulations show that linear interpolation is helpful, potentially with a variant of one of these tasks.

-The lack of replication of the prediction error result with the lottery task is interesting. The extra complexity of this version makes sense as a possible reason. Any timing differences between the designs should also be noted including the duration of any delays and time between trials. Average risk taking in the gain, mixed, and loss trials should also be reported because there could be significant behavioral differences to what has been observed in previous studies. It should also be noted in the discussion that ratings are collected after every trial in this study, and this is a key difference compared to much of the literature where ratings are not collected on every trial. Asking about mood on every trial may encourage participants to rely more on memory of the most recent event, and not the overall cumulative impact of expectations for multiple trials.

-Simulations do not seem to have any noise in them. It would be useful to have simulations with realistic amounts of noise (estimated from the actual data) to see how this impacts key findings, including parameter reliability for the distribution of parameter estimates observed from the data.

-The importance of controlling the precise feedback sequence does not come across clearly. Particularly since it doesn’t seem to make a difference for the results, it would be useful to make clear whether any simulations suggest that it would be valuable given what has been learned here.

Minor comments

-The opening sentence of the abstract refers to mood as being about outcomes related to intended outcomes. It is unclear what ‘intended outcomes’ means because presumably mood could change due to events that don’t have intended outcomes, like when someone reads the news.

-Mood ratings are said to be bounded by -1 and 1, but figure 4 shows z-scored ratings and then mean mood that is bounded by 0 and 1. It should be clear in all places whether analyses are about z-scored mood and when mood is not transformed and what the bounds are.

-Is wt for time the same as the drift in the paper by Jangraw that uses lottery tasks? The connection between what is found here and that paper should be discussed.

-In the table 2 caption and potentially elsewhere, it would be helpful to name each of the parameters so that readers are not confused about which one is for drift, for example. These should also be compared to published studies to the extent possible. Are these w0 the same as previously observed? These values are consistent with a -1 to 1 scale but a previous figure shows mean values that seem to be on a 0 to 1 scale. They should also be compared across the different tasks. For example, the forgetting factor seems to differ.

-Are the parameters correlated across tasks in participants that have completed two tasks? The means suggest differences, but the correlations across task should be included. Especially since w0 is not correlated in time in gambling but is for the other tasks, a careful comparison of w0 across tasks collected in the same session would be useful to understand why this parameter seems to be more stable in some tasks.

**********

**Have the authors made all data and (if applicable) computational code underlying the findings in their manuscript fully available?**

The PLOS Data policy requires authors to make all data and code underlying the findings described in their manuscript fully available without restriction, with rare exception (please refer to the Data Availability Statement in the manuscript PDF file). The data and code should be provided as part of the manuscript or its supporting information, or deposited to a public repository. For example, in addition to summary statistics, the data points behind means, medians and variance measures should be available. If there are restrictions on publicly sharing data or code —e.g. participant privacy or use of data from a third party—those must be specified.

Reviewer #1: None

Reviewer #2: **No:**  The gitbub link is not working

Reviewer #3: Yes

**********

PLOS authors have the option to publish the peer review history of their article (what does this mean?). If published, this will include your full peer review and any attached files.

If you choose “no”, your identity will remain anonymous but your review may still be made public.

**Do you want your identity to be public for this peer review?**  For information about this choice, including consent withdrawal, please see our Privacy Policy.

Reviewer #1: No

Reviewer #2: No

Reviewer #3: No

[NOTE: If reviewer comments were submitted as an attachment file, they will be attached to this email and accessible via the submission site. Please log into your account, locate the manuscript record, and check for the action link "View Attachments". If this link does not appear, there are no attachment files.]

**Figure resubmission:**

While revising your submission, we strongly recommend that you use PLOS’s NAAS tool (https://ngplosjournals.pagemajik.ai/artanalysis) to test your figure files. NAAS can convert your figure files to the TIFF file type and meet basic requirements (such as print size, resolution), or provide you with a report on issues that do not meet our requirements and that NAAS cannot fix.

After uploading your figures to PLOS’s NAAS tool - https://ngplosjournals.pagemajik.ai/artanalysis, NAAS will process the files provided and display the results in the "Uploaded Files" section of the page as the processing is complete. If the uploaded figures meet our requirements (or NAAS is able to fix the files to meet our requirements), the figure will be marked as "fixed" above. If NAAS is unable to fix the files, a red "failed" label will appear above. When NAAS has confirmed that the figure files meet our requirements, please download the file via the download option, and include these NAAS processed figure files when submitting your revised manuscript.

**Reproducibility:**

To enhance the reproducibility of your results, we recommend that authors of applicable studies deposit laboratory protocols in protocols.io, where a protocol can be assigned its own identifier (DOI) such that it can be cited independently in the future. Additionally, PLOS ONE offers an option to publish peer-reviewed clinical study protocols. Read more information on sharing protocols at https://plos.org/protocols?utm_medium=editorial-email&utm_source=authorletters&utm_campaign=protocols

# Author comment: Author response to Decision Letter 1

**DOI:** 10.1371/journal.pcbi.1014597.r002

26 Jun 2026

Attachment Submitted filename: Response to reviewers.docx

# Peer review: Decision Letter 1

**DOI:** 10.1371/journal.pcbi.1014597.r003

**Authors:** Zhaolei Zhang, Christoph Mathys

20 Jul 2026

Dear Dr. Benhamou,

We are pleased to inform you that your manuscript 'Assessing the validity and reliability of computational phenotyping of mood' has been provisionally accepted for publication in PLOS Computational Biology.

Before your manuscript can be formally accepted you will need to complete some formatting changes, which you will receive in a follow up email. A member of our team will be in touch with a set of requests.

Please note that your manuscript will not be scheduled for publication until you have made the required changes, so a swift response is appreciated.

IMPORTANT: The editorial review process is now complete. PLOS will only permit corrections to spelling, formatting or significant scientific errors from this point onwards. Requests for major changes, or any which affect the scientific understanding of your work, will cause delays to the publication date of your manuscript.

Should you, your institution's press office or the journal office choose to press release your paper, you will automatically be opted out of early publication. We ask that you notify us now if you or your institution is planning to press release the article. All press must be co-ordinated with PLOS.

Thank you again for supporting Open Access publishing; we are looking forward to publishing your work in PLOS Computational Biology.

Best regards,

Christoph Mathys

Academic Editor

PLOS Computational Biology

Zhaolei Zhang

Section Editor

PLOS Computational Biology

***********************************************************

Reviewer's Responses to Questions

**Comments to the Authors:**

**Please note here if the review is uploaded as an attachment.**

Reviewer #1: The authors addressed all my comments.

Reviewer #2: I would like to thank the authors for their responses to my comments. I believe the revisions have significantly improved the article - the text now reads more smoothly, the content is more comprehensive, and the results are presented and explained with greater clarity. I have no further comments to add.

Minor correction:

- line 247: "Recovery remained high for \omega"

Reviewer #3: The authors have done a good job of addressing the issues that I raised. I have no further comments. This is a great contribution to the literature and addresses a number of important questions in this growing area of research.

**********

**Have the authors made all data and (if applicable) computational code underlying the findings in their manuscript fully available?**

The PLOS Data policy requires authors to make all data and code underlying the findings described in their manuscript fully available without restriction, with rare exception (please refer to the Data Availability Statement in the manuscript PDF file). The data and code should be provided as part of the manuscript or its supporting information, or deposited to a public repository. For example, in addition to summary statistics, the data points behind means, medians and variance measures should be available. If there are restrictions on publicly sharing data or code —e.g. participant privacy or use of data from a third party—those must be specified.

Reviewer #1: None

Reviewer #2: Yes

Reviewer #3: Yes

**********

PLOS authors have the option to publish the peer review history of their article (what does this mean?). If published, this will include your full peer review and any attached files.

If you choose “no”, your identity will remain anonymous but your review may still be made public.

**Do you want your identity to be public for this peer review?**  For information about this choice, including consent withdrawal, please see our Privacy Policy.

Reviewer #1: No

Reviewer #2: No

Reviewer #3: No

# Editor report: Acceptance letter

**DOI:** 10.1371/journal.pcbi.1014597.r004

**Authors:** Zhaolei Zhang, Christoph Mathys

PCOMPBIOL-D-25-02233R1

Assessing the validity and reliability of computational phenotyping of mood

Dear Dr Benhamou,

I am pleased to inform you that your manuscript has been formally accepted for publication in PLOS Computational Biology. Your manuscript is now with our production department and you will be notified of the publication date in due course.

The corresponding author will soon be receiving a typeset proof for review, to ensure errors have not been introduced during production. Please review the PDF proof of your manuscript carefully, as this is the last chance to correct any errors. Please note that major changes, or those which affect the scientific understanding of the work, will likely cause delays to the publication date of your manuscript.

Soon after your final files are uploaded, unless you have opted out, the early version of your manuscript will be published online. The date of the early version will be your article's publication date. The final article will be published to the same URL, and all versions of the paper will be accessible to readers.

For Research, Software, and Methods articles, you will receive an invoice from PLOS for your publication fee after your manuscript has reached the completed accept phase. If you receive an email requesting payment before acceptance or for any other service, this may be a phishing scheme. Learn how to identify phishing emails and protect your accounts at https://explore.plos.org/phishing.

Thank you again for supporting PLOS Computational Biology and open-access publishing. We are looking forward to publishing your work!

With kind regards,

Sharmila Kamatchi

PLOS Computational Biology | Carlyle House, Carlyle Road, Cambridge CB4 3DN | United Kingdom ploscompbiol@plos.org | Phone +44 (0) 1223-442824 | ploscompbiol.org | @PLOSCompBiol
